# Characterizing choice behavior in *Drosophila melanogaster* olfactory classical conditioning

**DOI:** 10.1242/jeb.253164

**Published:** 2026-10-13

**Authors:** Samuel G. Stea, Tariq M. Brown, Rebecca Oramas, John S. Hernandez, Reza Azanchi, Karla R. Kaun

**Affiliations:** ^1^ Division of Biology and Medicine Biotechnology Graduate Program, Brown University, Providence, RI 02912, USA; ^2^ Department of Neuroscience, Brown University, Providence, RI 02912, USA; ^3^ Department of Neuroscience, Brown University, Providence, RI 02912, USA

**Keywords:** *Drosophila melanogaster*, Decision making, Choice, Memory, Classical conditioning, Pavlovian

## Abstract

*Drosophila melanogaster* is a powerful model organism to understand the neural and molecular mechanisms underlying learning and memory. However, memory is usually studied by assessing choice behavior at only one point in time in *D. melanogaster*, which limits our understanding of how flies respond to a learned cue. We developed a simple open-field-style assay that records the continuous behavioral dynamics of flies as they form preferences between two competing olfactory stimuli. We trained flies to associate odors with either a reward (sucrose) or a punishment (electric shock) and then placed them in a circular arena where they encountered a choice between a paired or unpaired odor. Within 30 s, flies approached the sugar-associated odor or avoided the shock-associated odor. Their collective behavior dynamics suggest the flies became more decisive over time; they traveled more on the preferred side, stayed closer to the source of the preferred cue, made more stops there, and consistently turned away from the alternative cue. We confirmed these results by showing that a learning-deficient mutant strain displayed no choice behavior. This assay provides enhanced resolution for understanding decision making as a continuous, dynamic process in *D. melanogaster*.

## Teaser

**Summary:** A novel open-field assay of olfactory learning and memory describes locomotor dynamics and discrete choice-related behaviors during expression of memory-based decision making in *Drosophila.*

## INTRODUCTION

The ability of an animal to recall cues associated with aversive stimuli, such as predators, or appetitive stimuli, such as food, enhances survival in complex and dynamic environments (Dukas, 2007; Shettleworth, 2009; Redish and Mizumori, 2015; Morand-Ferron, 2017). This highly conserved ability has been demonstrated in evolutionarily diverse animals, from sea slugs to humans (Walters et al., 1981; Molchan et al., 1994). Use of *Drosophila melanogaster* in particular has helped uncover conserved genetic and circuit-level components of associative memory during formation, maintenance and suppression (Davis, 1993, 2011, 2023; Dubnau et al., 2001, 2003; Heisenberg, 2003; Schwaerzel et al., 2003; Waddell, 2013; Aso et al., 2014; Cognigni et al., 2018).

The association between an odor and a reinforcer is the most well-characterized form of memory in *D. melanogaster* and remains a benchmark of learning and memory in contemporary research (Quinn et al., 1974; Tabone and de belle, 2014). In particular, the introduction of a simple T-maze assay in which flies chose between two odor options to avoid the one previously paired with intermittent shock marked a significant advancement in the field (Tully and Quinn, 1985; Pitman et al., 2009). The use of population-based memory assays, including the *D. melanogaster* T-maze, has accelerated the discovery of genes, signaling pathways and neural circuits involved in encoding aversive and appetitive memory (Dudai et al., 1976; Dudai, 1983; Dévav et al., 1986; Folkers et al., 1993; Schwaerzel et al., 2003; Guven-Ozkan and Davis, 2014; Lee, 2015; Boto et al., 2020; Davis, 2023). The widespread adaptation of these assays has also brought efforts to standardize training and testing protocols (Hay and Crossley, 1977; Krashes and Waddell, 2011a,b; Okray et al., 2025). Consequently, *D. melanogaster* olfactory conditioning methodology has remained largely unchanged for over 30 years (Dwijesha et al., 2024).

Decision making is a dynamic process (Wang, 2012; van Breugel, 2021; Harris and Hutcherson, 2022), yet maze-based assays typically restrict analysis of choice behavior to the distribution of an entire population at a single point in time, which limits the ability to understand the decision-making process. It is essential to measure decision making as it occurs, as distinct patterns of locomotion and transitions between stimuli occur across different stages of memory (Chabaud et al., 2010; Ichinose and Tanimoto, 2016). However, current olfactory conditioning assays cannot measure behaviors that individual flies within a group perform while choosing between a paired and an unpaired odor, leaving the behavioral strategies *D. melanogaster* exhibit during and after deciding poorly understood.

To better characterize choice-dependent behaviors in flies expressing associative olfactory memory, we introduce a high-throughput two-choice assay that consists of an open-field-style circular arena in which groups of flies can continuously choose between two odors streaming from opposite sides of the arena. For scalability, the apparatus consists of four circular arenas capable of testing multiple groups simultaneously, while also recording the data of individual animals over time to capture individual variation in choice behavior. During assessment of olfactory memory 2 min after training, we compared locomotor and positional metrics between arena sides during continuous access to conditioned stimuli. Here, we describe the time line of decision making between stimuli following appetitive (sucrose) and aversive (shock) conditioning, as well as choice-dependent behavioral metrics that may better characterize decision making in *D. melanogaster*.

## MATERIALS AND METHODS

### Fly husbandry

The following *Drosophila melanogaster* strains were used for appetitive and aversive short-term memory experiments: *Canton-S* (Bloomington Drosophila Stock Center, RRID:BDSC_64349), *w^1118^* (RRID:BDSC_3605) and *dunce* (Pde4[dnc-1], RRID:BDSC_6020). Flies were reared on standard cornmeal–agar food with tegosept antifungal and propionic acid antibacterial substrates inside 40 ml plastic vials and housed in a 24°C incubator at 70% humidity with a 14 h:10 h light:dark cycle with lights on at 07:00 h. Vials were flipped every 3 days to prevent crowding.

Male flies were used for all experiments, as we have previously reported sex differences in appetitive and aversive odor exploration (Hernandez et al., 2025), and we plan to investigate sex differences in a future study. Males, 1–2 days old, were collected under humidified CO_2_ for <5 min and given 24 h to recover. Following CO_2_ recovery, all flies underwent 24 h of wet starvation via placement in 10 ml vials prefilled with 2 ml of 1% agar to homogenize hunger state prior to training. Vials housed 30 collected flies to ensure 20 flies per vial could be tested following starvation. To avoid the confounding effects of CO_2_ anesthesia on behavioral metrics during training and testing, flies were transferred after CO_2_ recovery using an aspirator, a device used to collect flies that are not anesthetized. All flies were 3–5 days old at the time of testing. Behavior tests were conducted between 2 and 9 h after lights on.

### Behavior environment

All behavior experiments were conducted inside a dark temperature- and humidity-controlled environment. The chamber consisted of a 68×53×43 cm^3^ box composed of 5 mm thick black corrugated plastic boards (CorrugatedPlastics.Net) with an accessible entrance at the front that, when closed, provided a dark environment. A SMART 200 W electric flat panel radiant heating plate with an adjustable thermostat (SUMLGT, Shenzhen Jinhui Technology Co., Ltd) and two attachable 12×12×2.5 cm 12 V low-noise 3-Pin 1400 rpm computer fans (UMLIFE) was placed at the back of the box. Humidity was controlled by flowing humidified air directly into the circular behavior arenas via Teflon tubing. During operation, the environment within the box was recorded as having a temperature of 22.9±0.6°C and a 64±12% humidity. A circular port at the top of the box fed directly to an exhaust arm to prevent odor accumulation within the box.

### Two-choice assay components and fabrication

All components for the assay and airflow equipment are listed in Table S1. The two-choice apparatus consisted of stacked Teflon (CS Hyde, Lake Villa, IL, USA) and white acrylic (Acrylic-TL-White-7328, Plastics 2000, Modesto, CA, USA) sheets that form a four-arena behavioral chamber (Figs S1 and S2A). Two removable clear Plexiglas lids (TAP Plastics) were used to cover the arenas. A PDF schematic for laser-cutting components of the apparatus is available from GitHub (https://github.com/kaun-lab). Each circular arena contained two port entrances on opposite sides for simultaneous introduction of odors. Nylon mesh port screens cut to 6×2 mm rectangles (McMaster-Carr, Elmhurst, IL, USA) were fitted within slits covering the port entrances. Two perforated parallel lines surrounding the midline in each circular arena allowed the vacuuming of air or odor to prevent odor accumulation over time. Sheets were glued (Loctite, 681925) and lids fastened with nylon socket head screws (95868A261, McMaster-Carr). Teflon tubes (3 mm diameter, F015090, Fluorostore, Parsippany, NJ, USA) were pushed into each entrance and secured. Tubes and screens were covered with PTFE FEP tape (CS Hyde). Teflon tubes were capped with 1/8 inch male luer-to-hose barb adapters (MFLX30800-24, Masterflex, Vernon Hills, IL, USA) for connection to odor source tubes. The vacuum tube was inserted and secured in the vacuum chamber of the two-choice arenas.

### Air and odor delivery

Two air and one vacuum flowmeter were set at 50 and 150 ml min^−1^, respectively, on flowmeters (air flowmeters 112-02 Glass; vacuum flowmeter 092-04 Glass, Cole-Parmer, Vernon Hills, IL, USA), such that each 50 ml min^−1^ of air flowed into both ports of an arena at once and would not build up within the arenas during tests (Fig. S2B). A three-way valve (411L3212HV, Asko, Suwanee, GA, USA) was connected via swivel-elbow tubes (7880T189, McMaster-Carr) to a flowmeter (Cole-Parmer) and two 40 ml glass vials with polypropylene caps (Fisher), one filled with 3 ml odor and the other with 10 ml ddH_2_O. Two plastic luer lock tube couplings (51525K222, McMaster-Carr) were tapped into each odor vial cap and connected to Teflon tubes leading to each arena. A custom switchboard was wired to a three-way valve to control the airflow to vials with ddH_2_O or odors. The tube exiting the ddH_2_O vial and the tube exiting the odor were joined into a single tube by a Wye connector (5111K542, McMaster-Carr), and the exiting single tube was connected to another Wye connector to lead the lines to the two ports of the arena. Each of the airflow meters supplied air to the connected odor vials.

Ethyl acetate (EA; Fisher Science Education, Hanover Park, IL, USA) and isoamyl acetate (IAAc; Fisher Science Education) were chosen as odors because they are present in multiple fruit species that are appetitive to *D. melanogaster* (Dukas, 1999). Odors were diluted in mineral oil (Fisher Science Education). After dilution, 3 ml of each odor solution was pipetted into the glass vials attached to the two-choice tubes.

### Odor–shock training

Shock arenas were composed of layered acrylic materials as described in Scaplen et al. (2019) with the addition of a custom-designed copper grid with 36, 0.5 mm-wide copper (BasicCopper) strips spaced 0.5 mm apart fixed to the arena floor (Fig. S3). A PDF schematic for laser cutting each shock arena layer and the Plexiglas lids is available from GitHub (https://github.com/kaun-lab). Connecting wires from the copper grid were attached to each arena floor and fed into a single-pin cable mount (Switchcraft, Chicago, IL, USA) leading to an S48 square pulse stimulator, (Astro-Med, Warwick, RI, USA). To keep the training environment as similar as possible to the testing environment, the dimensions of the shock arena were made identical to those of the two-choice arenas.

To control for the impacts of food deprivation on memory formation and ensure similar baseline motivational states for appetitive odors during odor choice, all shock-trained flies received the same starvation period prior to training as sucrose-trained flies. Flies were wet-starved for 24 h via placement in 10 ml vials prefilled with 2 ml of 1% agar. To condition shock memory, flies within agar vials were taken from the incubator and aspirated into shock arenas in the dark at 24°C and 70% humidity and exposed to either EA (1:512) or IAAc (1:1024) without shock for 2 min. Flies were then exposed to the reciprocal odor while receiving 80 V shocks from the pulse stimulator lasting 1.5 s every 5 s for 2 min (Fig. S2C). Because shock conditioning took place in arenas with the same dimensions as the two-choice assay, the same flow rates were used (50 ml min^−1^). The 2 min exposures to each odor and condition were repeated a total of three times in one training session.

### Odor–sucrose training

Sucrose training vials consisted of two 40 ml clear polypropylene tubes (Fisher) containing a filter paper dampened with 3 ml of either pure ddH_2_O or 2 mol l^−1^ sucrose dissolved in ddH_2_O as performed in previous olfactory conditioning studies (Kaun et al., 2011; Vogt et al., 2014). The ddH_2_O and sucrose filter papers were lined along the inner perimeter of the training vials the previous evening and left to dry overnight. Odor exposure occurred by placing training vials into one of two 1280 cm^3^ Plexiglas boxes containing either EA or IAAc (Kaun et al., 2011). Training vials included a rubber cap with a 24 mm diameter circular hole covered with nylon mesh screen to expose flies to odor stimuli within the Plexiglas boxes. Because the volume of each Plexiglas box was much greater than that of the shock-training and two-choice arenas, odor flow rates to each box were set at 1.2 l m^−1^ by flowmeters (092-04 Glass, Cole-Parmer). Airflow proceeded from 1/2 inch inner diameter Teflon tubing (5239K17, McMaster-Carr) to a glass bubbler containing 200 ml ddH_2_O. Humidified air reached both glass vials containing each odor simultaneously by forking into separate tubes, then flowed to each Plexiglas box. A passive vacuum port at the opposite end of each box directed the air/odor out at the same flow rate as the incoming air/odor.

All sucrose-trained flies were wet-starved for 24 h by being placed in 10 ml vials prefilled with 2 ml of 1% agar. To condition sucrose memory, flies were brought from the incubator to a Plexiglas box set to 24°C and 70% humidity and gently tapped into training vials containing H_2_O-dampened filter paper in the presence of an odor. Following 2 min of odor exposure in the H_2_O training vial, the vial was inverted to transfer flies into the second training vial containing filter paper dampened with 3 ml of 2 mol l^−1^ sucrose in ddH_2_O and exposed to the reciprocal odor in the second Plexiglas box for 2 min (Fig. S2D). The 2 min exposures to each odor and condition were repeated a total of three times with no breaks between exposures in one training session. Sucrose training occurred under red light to allow the experimenter to see as vials were transferred between training boxes.

### Odor-choice test

Two minutes after shock or sucrose memory training, flies were aspirated into the two-choice apparatus (10 flies per arena). A single genotype/condition was tested in two arenas in each test. Each memory test lasted 135 s; flies received only air for the first 15 s (henceforth referred to as the ‘odor-off’ phase), then were exposed to EA and IAAc simultaneously from opposite ports for the remainder of the test.

To identify the time period when exploration ended and odor choice occurred, we averaged arena side transitions in 5 s bins over time as a previously used metric of choice (Chabaud et al., 2010) in naive *Canton-S* flies. Transitions were counted as the number of instances a fly's center of mass entered the opposite arena side. We found that transitions decreased when comparing the bins at 5 and 30 s following odor exposure but not during the remaining 90 s (Fig. 1A–D). This remained true when transitions were reanalyzed in 1 s bins (Fig. S4) and suggested a collective odor choice occurred in the final 90 s of the test. Thus, these 25 and 90 s time periods are referred to as the ‘exploration’ and ‘choice’ phases, respectively. We used these phases to quantify odor preference by comparing average preference during odor choice (45–135 s) with preference during the odor-off (0–15 s) phase on the two-choice assay. The first 5 s of odor exposure were not included in the exploration phase to ensure that this phase captured behavior only after odors had had sufficient time to fully permeate the arena.

**Fig. 1.**
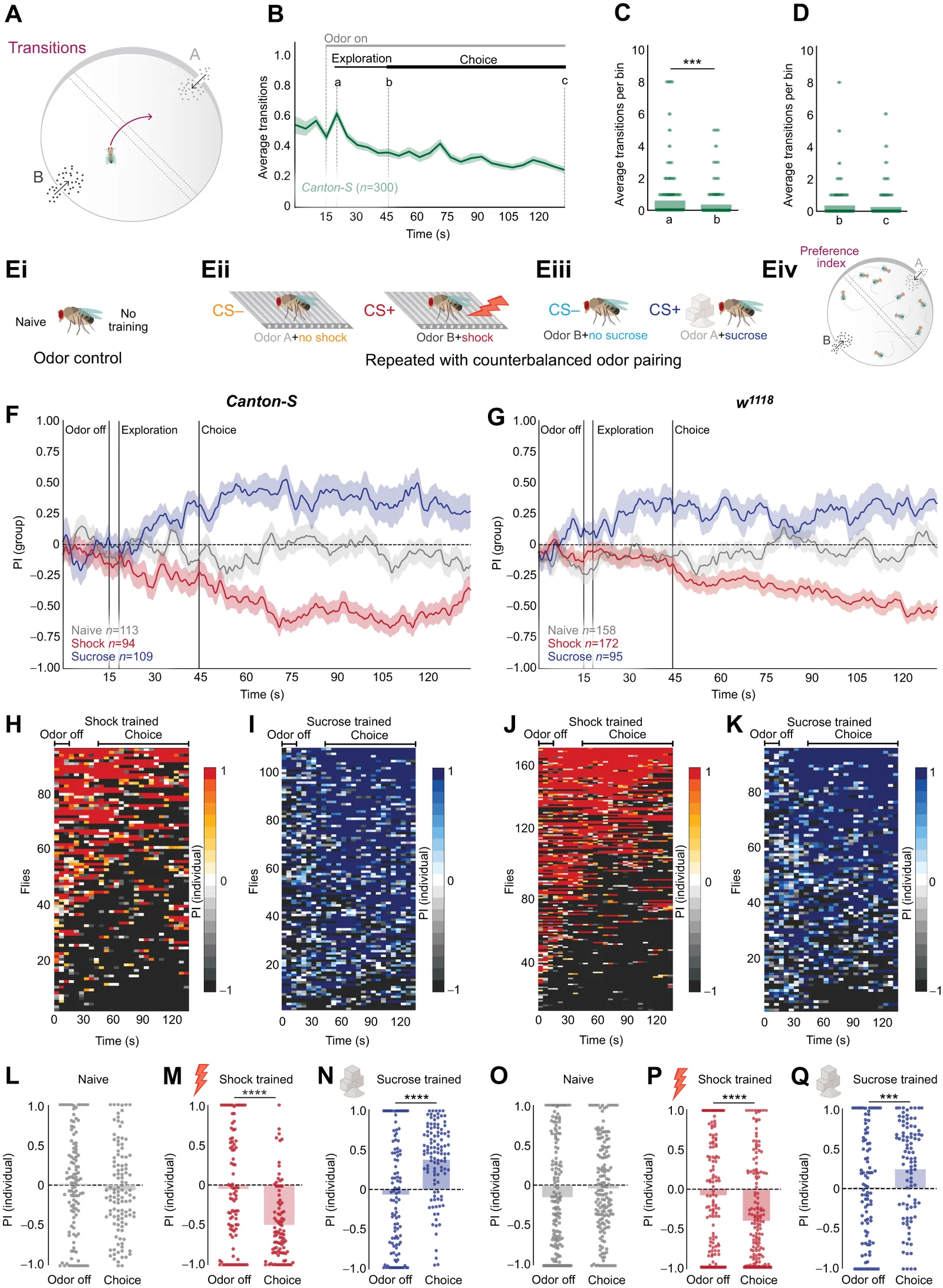
**Olfactory memory in *Canton-S* and *w^1118^ Drosophila melanogaster*.** Line graphs depict means±s.e.m. Bar graphs depict means with each dot representing values for a single fly. (A) Graphic of arena side transitions. (B) Comparing mean transitions per 5 s bin at different times of the test suggests distinct phases of exploration and choice. (C,D) Compared with the bin that began 5 s after odor exposure (labeled as bin a), mean transitions decreased 30 s after odor exposure (bin b) (C); compared with bin b, mean transitions did not decrease in the final bin of the test that began 90 s after odor exposure (bin c) (D). (E) Graphic of the training conditions and preference index (PI) used to assess memory: (i) control; (ii) odor–shock; (iii) odor–sucrose. The per-group PI visualizes average preference continuously over time (iv). A positive PI in trained groups indicates a preference for the conditioned stimulus (sucrose or shock). The PI index for naive flies is plotted relative to arena side A. *Canton-S* (F) and *w^1118^* (G) flies show increased mean group preference for the sucrose-paired odor and decreased preference for the shock-paired odor following the first 30 s of odor exposure. (H–K) PI per fly, averaged across frames for *Canton-S* (H,I) and *w^1118^* (J,K), depicts trends in preference over time across individual flies following shock or sucrose training. (L–Q) Memory was quantified by comparing the mean individual PI between the odor-off and choice phases. (L) Naive *Canton-S* flies did not develop a side preference by the post-choice period, regardless of which odor was presented on each side. (M) Shock-trained *Canton-S* flies avoided the shock-paired odor. (N) Sucrose-trained *Canton-S* flies preferred the odor paired with sucrose. (O) Naive *w^1118^* flies did not develop a side preference. (P) Shock-trained *w^1118^* flies avoided the shock-paired odor. (Q) Sucrose-trained *w^1118^* flies preferred the sucrose-paired odor. Asterisks denote significant differences (***P<0.001, ****P<0.0001). Samples, statistical tests and detailed results are given in Table S2. Experimental replicate and fly exclusion details are given in Table S4.

All tests for a respective genotype/condition took place on the same day, and the odor paired with the unconditioned stimulus (sucrose or shock) was switched between each test to reduce potential odor bias. Arenas were wiped down with ddH_2_O and dried between each test, and odor vials were replaced every other test to ensure consistent potency. Naive controls did not receive odor conditioning before testing.

### Odor concentration selection

To identify concentrations of odors that did not elicit a natural bias to either odor when presented simultaneously (which could confound memory expression), we exposed naive flies to different concentrations of EA or IAAc and compared their collective side preference between the odor-off and choice phases (Fig. S5A). Prior to these tests, flies were wet-starved for 24 h via placement in 10 ml vials prefilled with 2 ml of 1% agar to duplicate the starvation conditions of flies that would undergo olfactory conditioning. The pairs of concentrations evaluated were 1:512 EA, 1:512 IAAc; 1:1024 EA, 1:1024 IAAc; and 1:512 EA, 1:1024 IAAc (Fig. S5B–G). Concentrations of 1:512 EA and 1:1024 IAAc presented simultaneously did not invoke a biased preference in naive *Canton-S* or *w^1118^* flies (Fig. S5F–I) and were used in subsequent memory tests.

### Data collection and statistical analysis

Videos were recorded at 30 frames s^−1^ and saved as 8-bit grayscale .AVI files using FlyGrAM software and an arena stand (Scaplen et al., 2019). In the FlyGrAM GUI, the ‘Experimental Duration’ parameter was set to 135 s, the ‘Time until Stimulus Onset’ parameter was set to 15 s, and the ‘Stimulus Duration’ parameter was set to 120 s. ‘Write .AVI Video’ and ‘Write .CSV File’ checkboxes were also selected. Recordings were captured with a USB camera (USBFHD01M-L21, ELPCCTV) capped with a 6 mm focal length F2.0, M12 adaptor lens (PT-0620, M12 Lenses, Maitland, FL, USA). An infrared filter [54–664 (RG-850), Edmund Optics, Barrington, NJ, USA] was applied to the lens to record tests while avoiding the confounding effects of light exposure (Han et al., 2025). Raw data were retrieved from video files using TRex software (Walter and Couzin, 2021).

Individual flies were identified by setting a pixel darkness threshold for detection within TRex. Within the standard 8-bit pixel intensity scale of 0–255, with a value of zero representing a black pixel and a value of 255 representing a white pixel, the TRex default threshold of 15 effectively distinguished pixels representing flies from the arena background. The position of each fly was represented as the center of mass or ‘centroid’ point in TRex, and the midline separating arena sides was defined by a 2-dimensional line, such that a fly could not be classified as part-way or ‘in between’ arena sides. Arena lengths for each video were manually converted from pixels to cm by indicating the boundaries of an arena with the use_known_length_to_calibrate command. If TRex lost track of a fly's position, their data were supplanted by the average of values directly before and after the period during which tracking was lost. The average number and percentage of frames lost across loss-of-tracking instances per fly are reported in the second row of Table S4 for each genotype and condition. While we opted to use default Trex settings for all video processing, TRex offers an additional output_recognition_data command that records recognition scores assigned to each fly for each frame. Recognition scores are generated from a convolutional neural network that determines the best visual features for predicting identity, and is trained by scoring variants of initial frames with known identities (Walter and Couzin, 2021). All raw behavioral metrics outputted by TRex were aggregated using a custom-written PANDAS script (annotated code and instructions available from GitHub: https://github.com/kaun-lab).

The behaviors of midline U-turns and stops close to the odor port adopted the parameters of a sharp turn and stop behavior described in Ctrax software (Branson et al., 2009) as well as the following additional parameters: U-turns began within 0.5 cm of the midline with the fly facing the midline (head at a <|90| deg angle from the midline) and ended with the head's orientation at a greater angle from the midline than at the beginning of the U-turn (Fig. S6). Stops close to the odor port occurred within 0.9 cm of the port, closer to the odor port than the arena midline.

To quantify discrete behaviors that do not occur continuously (midline U-turns, stops close to odor port and arena side transitions), the average number of each behavior made per fly was binned every 5 s before being averaged across flies. The bin size of 5 s was chosen to avoid reporting rates of discrete behaviors in trivially small units (e.g. average U-turns made per frame would be a small decimal), and the ability to visualize change over time was retained when plotted on a line graph. For this reason, we also chose to plot distance traveled over time in 5 s bins. Distance traveled was presented in two ways: (1) average regional distance traveled and (2) average cumulative distance traveled. Regional distance was calculated by separately averaging the distance traveled per frame across flies on each arena side, then binning per-frame averages every 5 s. Cumulative distance was calculated by averaging the total distance each fly traveled since the start of the experiment every 5 s.

Memory was quantified with a preference index (PI); the proportion of flies that avoided shock-paired odor (or approached the sucrose-paired odor) minus the proportion that did not (Quinn et al., 1974). To visualize both choice dynamics across time and variability across flies, PI was also calculated in two ways, by group (arenas containing 10 flies) and by individual fly. Line graphs depict average group (arena) PI over time, which was calculated at each frame by subtracting the number of flies on the unpaired odor side from the number of flies on the paired odor side and dividing by the total number of flies (Ichinose and Tanimoto, 2016). The group PI at frame *t* is:

(1) $$PI_{\mathrm{group}}(t)=\frac{N_{+}(t)-N_{-}(t)}{N_{+}(t)+N_{-}(t)},$$

where *N*_+_(*t*) and *N*_−_(*t*) denote the number of flies occupying arena sides containing the odor that was paired or not paired with the conditioned stimulus in one frame, respectively.

Bar graphs depict the average individual (fly) PI, with individual flies represented by dots. To determine individual PI values per fly, the time a fly spent on the unpaired odor side was subtracted from the time spent on the paired odor side out of a total time interval of interest (*T*), expressed as:

(2) $$PI_{f}=\;\frac{1}{T}\overset{T}{\underset{t=0}{\sum }}F_{f}\;(t),$$

where *F_f_*(*t*) indicates the PI value of each fly (*f*) across each frame (*t*):

(3) $$F_{f}\;(t)=\;\{\begin{array}{c} +1\;\mathrm{if}\;\mathrm{in}\;\mathrm{CS}+\\ -1\;\mathrm{if}\;\mathrm{in}\;\mathrm{CS}- \end{array}$$

and averaged across individuals:

(4) $${\bar{\mathrm{PI}}}_{\mathrm{indiv}}=\frac{1}{N}\overset{N}{\underset{\;f=1}{\sum }}PI_{f}.$$

Flies that did not make at least one transition across arena sides during the trial were excluded for all measures, including group and individual PI, and were not included in the final calculated averages. All included flies must therefore have an individual PI of >−1 and <1 when calculated over the entire trial. However, as individual PI was calculated over a portion of a trial, such as before odor introduction, included flies may still have a value of −1 or 1 for these time frames, indicating that they did not transition within that part of the behavior experiment, but did so at a different time in the trial. The number and percentage of flies excluded per group are reported in the last column of Table S4.

To ensure sufficient statistical power, a minimum of eight arenas (10 flies per arena) were tested for each condition/genotype, allowing within-group analyses to reflect at least eight independent tests. All *N* counts and statistical tests per figure are provided in Tables S2 and S3, and arena replicates per condition are provided in column 4 of Table S4. Statistical tests were performed in GraphPad Prism 10.5.0. Assumptions of normality were assessed with the Shapiro–Wilk test for paired and unpaired *t*-tests and with the D'Agostino–Pearson omnibus test for ANOVA. Homogeneity of variance was determined using Levene's test for unpaired *t*-tests and ANOVA. Two-way ANOVA compared effects of (arena side×experimental phase), with experimental phases of ‘odor-off’, ‘exploration’ and ‘choice’ defined in the ‘Odor-choice test’ section above as the intervals of 0–15, 20–45 and 45–135 s, respectively. One-way ANOVA compared effects between training conditions, and *post hoc* pairwise comparisons following ANOVA were evaluated by Tukey's multiple comparisons. Within each condition, comparisons between arena sides and between experimental phases were performed using paired *t*-tests. Comparisons between genotypes were performed using unpaired *t*-tests. Principal component analysis (PCA) was performed using Prism on the standardized data of all metrics averaged per fly (individual PI, transitions, bout duration, distance traveled, U-turns, port distance and stops close to port). The option to standardize data was selected to avoid skewing caused by differing units of each metric. Parallel analysis was used to select principal components.

All data files and recordings are available for download in the Brown Digital Repository (https://doi.org/10.26300/nwk1-jy27).

## RESULTS

### A simple two-choice assay to test olfactory memory

We first assessed the temporal dynamics of decision making in the two-choice apparatus by quantifying the number of transitions *Canton-S* flies made between two equally preferred odors throughout a 2 min period (Fig. 1A,B; Figs S4A,B and S5). We noted that flies significantly reduced the number of transitions between the two bins at 5 and 30 s after odors were introduced (Fig. 1C, *P*=0.0007; Fig. S4C), and while they continued to transition between odor sides, the number of transitions did not change when comparing the bins at 30 and 90 s after odors were introduced (Fig. 1D, *P*=0.06, Fig. S4D). These results suggested that flies exhibit distinct phases of odor exploration and choice (Fig. 1B) and that olfactory memory may also be observed by quantifying preference in the choice phase.

We then assessed memory for an odor associated with shock or sucrose in two genetic backgrounds commonly used in memory assays (*Canton-S* and *w^1118^*). We trained flies to associate an odor with three successive 2 min sessions of 80 mV shock, or three successive 2 min sessions of 2 mol l^−1^ sucrose (Fig. 1E). Naive flies did not receive any previous experience with odors, shock or sucrose. Following training, we evaluated whether flies would avoid an odor previously paired with shock or move towards an odor previously paired with sucrose in our two-choice assay.

To visualize memory, we first looked at the average preference index of groups of flies (group PI), where preference was calculated per arena by subtracting the number of flies on the unpaired odor side from that on the paired odor side and dividing by the total number of flies in the arena. Group PI was plotted continuously over time in *Canton-S* (Fig. 1F) and *w^1118^* flies (Fig. 1G). To determine memory replication across arenas, we plotted average PI per fly within each arena and found that flies within the arena tended to move away from the odor paired with shock and towards the odor paired with sucrose (Fig. S7), indicating that group memory depicted in Fig. 1F,G was driven by most flies successfully learning instead of a small proportion of strong learners.

To gain an understanding of individual variation in PI, we plotted time spent in the paired odor side per fly, noting that most flies will transition between the two odor choices throughout the experiment, and their accumulated time in each side reflected their preference (Fig. 1H–K). Most *Canton-S* flies trained to associate an odor with shock demonstrated an increasing amount of time spent avoiding the paired odor (Fig. 1H), whereas those trained to associate an odor with sugar showed an increasing amount of time preferring the paired odor (Fig. 1I). Similar trends were seen with *w^1118^* flies (Fig. 1J,K).

To quantify memory formation across individual flies (individual PI), we compared the average PI of flies between the odor-off phase (0–15 s) and choice phase (45–135 s). The average PI of naive *Canton-S* flies did not differ between odor-off and choice phases (Fig. 1L, *P*=0.19). However, shock-trained *Canton-S* flies exhibited greater avoidance of the shock-paired odor during the choice phase (Fig. 1M, *P<*0.0001), while sucrose-trained *Canton-S* flies exhibited greater preference for the sucrose-paired odor during the choice phase (Fig. 1N, *P<*0.0001).

Similar to their *Canton-S* counterparts, naive *w^1118^* flies did not develop an arena side preference (Fig. 1O, *P*=0.11). However, shock-trained *w^1118^* flies showed greater avoidance of the side containing the shock-paired odor (Fig. 1P, *P<*0.0001), and sucrose-trained *w^1118^* flies showed a greater preference for the side containing the sucrose-paired odor (Fig. 1Q, *P*=0.007). These results demonstrated robust aversive and appetitive olfactory memory in both wild-type *Canton-S* and *w^1118^* mutant flies.

To determine whether flies were forming associations between each odor and the unconditioned stimulus similarly, we assessed memory for stimuli paired with each odor individually (Fig. S8). Shock-trained *Canton-S* flies (Fig. S8A) avoided shock paired with either EA (Fig. S8B, *P<*0.0001) or IAAc (Fig. S8C, *P*=0.02). Shock-trained *w^1118^* flies (Fig. S8D) also avoided shock paired with either EA (Fig. S8E, *P*=0.02) or IAAc (Fig. S8F, *P*=0.0004). Sucrose-trained *Canton-S* flies that received sucrose paired with EA (Fig. S8G,H, *P<*0.0001) but not IAAc (Fig. S8I, *P*=0.22) preferred the sucrose-paired odor. In contrast, sucrose-trained *w^1118^* flies that received sucrose paired with IAAc (Fig. S8J,K, *P*=0.004) but not EA (Fig. S8L, *P*=0.18) preferred the sucrose-paired odor. This suggests that behavioral expression of memory may depend on the odors used in the assay and how they interact with both the unconditioned stimulus and the genetic background of the flies being tested.

To ensure odor choice was not influenced by odors, shock or sucrose exposure alone, we performed experiments (Fig. S9A) where flies received odor (Fig. S9B–E), sucrose (Fig. S9F–I) or shock (Fig. S9J–M) exposure for the same duration as they would have done during memory training, and tested odor choice. These prior experiences resulted in a general reduction in arena side transitions over time (Fig. S10), but did not change odor preference during the choice phase in either *Canton-S* or *w^1118^* flies (Fig. S9N), suggesting that odor choice in trained flies reflects associative memory expression. Reduced average transitions exclusively between the first and last 5 s bins of the exploration phase as an indicator of distinct behavioral phases was found in *w^1118^* but not *Canton-S* flies (Fig. S10A–F). Given that average transitions over time in naive *Canton-S* flies indicated distinct exploration and choice phases (Fig. 1B–D), the effect of individual stimuli across genetic backgrounds should be considered in future studies.

### Distance moved is associated with learned preference

Regional locomotor activity has previously been used to measure deliberate engagement and choice of conditioned stimuli in rodents (Totty et al., 2021; Iglesias et al., 2023) and *D. melanogaster* (Chabaud et al., 2010). We hypothesized that shock-trained flies would exhibit lower activity in the presence of shock-paired odor compared with the no-shock-paired odor (akin to freezing), and sucrose-trained flies would exhibit greater locomotor activity in the presence of sucrose-paired odor compared with the no-sucrose-paired odor. To evaluate this, we analyzed the mean distance traveled per 5 s bin on each arena side. To account for unequal distribution of flies over time, means were initially calculated per frame and normalized to the number of flies occupying each side before binning.

In naive flies, distance traveled did not differ between arena sides (Fig. 2A,B, *Canton-S P*=0.79; Fig. 2C,D, *w^1118^ P*=0.29). Shock-trained *Canton-S* flies traveled more on the no-shock-paired odor side during the odor-off phase (Fig. 2E,F, *P*=0.03), exploration phase (*P*=0.0002) and choice phase (*P<*0.0001). Shock-trained *w^1118^* flies also traveled more on the no-shock-paired odor side during the odor-off phase (Fig. 2G,H) (*P*=0.003), exploration phase (*P*=0.002) and choice phase (*P<*0.0001). While effects during the odor-off phase could have reflected a response to trace amounts of odor residue or an overall reduction in activity in shock-trained flies, differences were greatest in the choice phase for both genotypes, suggesting a distinct reduction in activity in the presence of the conditioned odor.

**Fig. 2.**
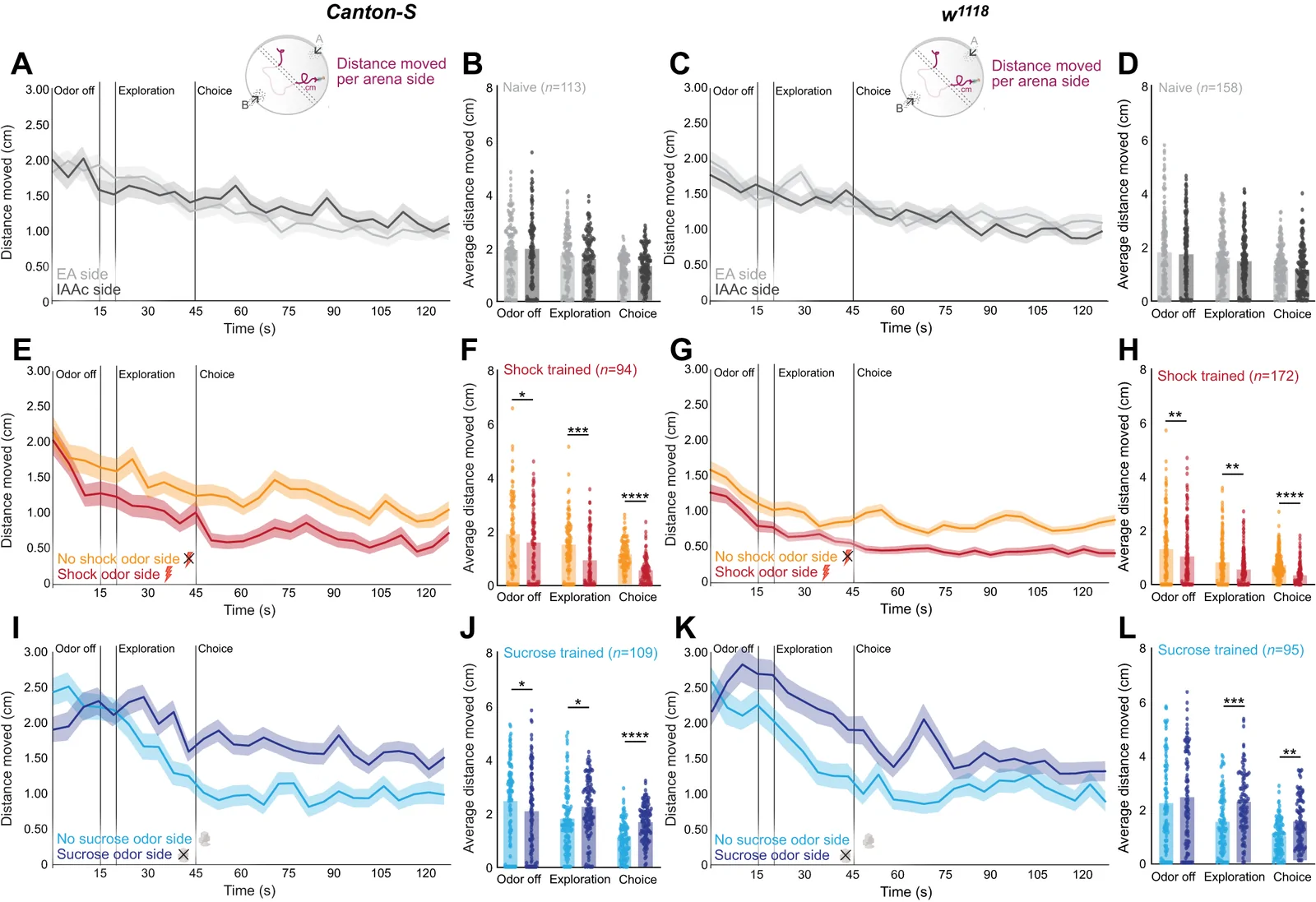
**Regional distance traveled reflects memory.** (A–L) Distance traveled per region was normalized to the number of flies occupying each region across each video frame. Line graphs depict mean±s.e.m distance traveled per 5 s time bin. Bar graphs depict mean distance traveled across each experimental phase and dots represent total distance traveled within a phase by each fly. Neither naive *Canton-S* (A,B) nor *w^1118^* (C,D) flies traveled more on either side over time. Shock-trained *Canton-S* (E,F) and *w^1118^* (G,H) flies traveled less on the side containing the shock-paired odor over time. Sucrose-trained *Canton-S* (I,J) and *w^1118^* (K,L) flies traveled more on the side containing the sucrose-paired odor. Asterisks denote significant differences (*P<0.05, **P<0.01, ***P<0.001, ****P<0.0001). Samples, statistical tests and detailed results are given in Table S2. Experimental replicate and fly exclusion details are given in Table S4. EA, ethyl acetate; IAAc, isoamyl acetate.

Following sucrose training, *Canton-S* flies traveled more on the no-sucrose-paired odor side during the odor-off phase (Fig. 2I,J) (*P*=0.02) but traveled more on the sucrose-paired odor side during the exploration phase (*P*=0.02) and choice phase (*P<*0.0001). *w^1118^* flies did not travel more on either side during the odor-off phase (Fig. 2K,L) (*P*=0.22) but traveled more on the sucrose-paired odor side during the exploration phase (*P*=0.0002) and choice phase (*P*=0.01). These results indicate that sucrose-trained flies traveled more on the side containing the sucrose-paired odor.

To test whether odor–shock or odor–sucrose training impacted locomotion, we compared the average distance traveled throughout the test (Fig. S11A,B). Shock-trained *Canton-S* flies traveled less overall than naive *Canton-S* flies (Fig. S11C, *P<*0.0001) but not during the odor-off phase (Fig. S11D, *P*=0.59). Shock-trained *w^1118^* flies traveled less overall than naive *w^1118^* flies (Fig. S11E, *P<*0.0001) and less during the odor-off phase (Fig. S11F, *P<*0.0001). In contrast, sucrose-trained *Canton-S* flies traveled more than naive *Canton-S* flies (*P<*0.0001) and more during the odor-off phase (*P*=0.002). Sucrose-trained *w^1118^* flies also traveled more overall than naive *w^1118^* flies (*P*=0.002) and more during the odor-off phase (*P*=0.04). These results show that sucrose training increased locomotor activity and shock training decreased activity. However, regional distance traveled depended on arena side in both training conditions, despite shock and sucrose training exerting opposite effects on locomotion. Overall, distinct locomotor responses in the presence of the conditioned odor reflect a learned behavioral response that may be expressed as the choice to engage with one stimulus over another.

### Bout duration is associated with learned preference

We hypothesized that learned preference would be demonstrated by a reduction in the number of transitions in trained flies during the choice period compared with naive flies. To measure this, we averaged the number of transitions between arena sides binned every 5 s (Fig. S12A–F). Compared with naive *Canton-S* flies, shock-trained *Canton-S* flies made fewer average transitions per bin in the choice phase (Fig. S12G, *P<*0.0001) and fewer overall transitions (Fig. S12H, *P<*0.0001). However, sucrose-trained *Canton-S* flies did not make fewer average transitions in the choice phase (Fig. S12G, *P*=0.99) or fewer total transitions (Fig. S12H, *P*=0.99) than naive flies. Shock-trained *w^1118^* flies made fewer average transitions per time bin in the choice phase (Fig. S12I, *P<*0.0001) and fewer overall transitions (Fig. S12J, *P<*0.0001) than naive *w^1118^* flies. Sucrose-trained *w^1118^* flies made fewer average transitions in the choice phase (Fig. S12I, *P*=0.99) and made fewer overall transitions (Fig. S12J, *P*=0.03). Given that sucrose-trained flies showed a clear preference for sucrose-paired odors (Fig. 1F,G), the lack of difference between sucrose-trained and naive flies in the number of transitions may simply reflect greater average locomotor activity in sucrose-trained flies (Fig. S11).

To further assess transitions as a metric of choice, we evaluated the average latency to transition from each arena side, hereafter referred to as ‘bout duration’ (Fig. 3). We hypothesized that over time, trained flies would wait longer to transition out of their preferred arena side. As predicted, average bout duration throughout the experiment did not differ between arena sides in naive flies (Fig. 3A,B, *Canton-S P*=0.45; Fig. 3C,D, *w^1118^ P*=0.94). Following shock training, there was no difference in bout duration between sides in either genotype during the odor-off phase (Fig. 3E,F, *Canton-S P*=0.29; Fig. 3G,H, *w^1118^ P*=0.26), but both genotypes had a reduced average bout duration on the shock-paired odor side during the exploration phase (*Canton-S P<*0.0001; *w^1118^ P*=0.008) and the choice phase (*Canton-S P<*0.0001; *w^1118^ P<*0.0001). Following sucrose training, *Canton-S* flies exhibited lower bout duration on the sucrose-paired odor side during the odor-off phase (Fig. 3I,J) (*P*=0.04), but greater bout duration on the sucrose-paired odor side during the exploration phase (*P*=0.006) and choice phase (*P<*0.0001). In sucrose-trained *w^1118^*, bout duration did not differ between sides during the odor-off phase (Fig. 3K,L) (*P*=0.67) but was greater in the sucrose-paired odor side during the exploration phase (*P<*0.0001) and choice phase (*P<*0.0001).

**Fig. 3.**
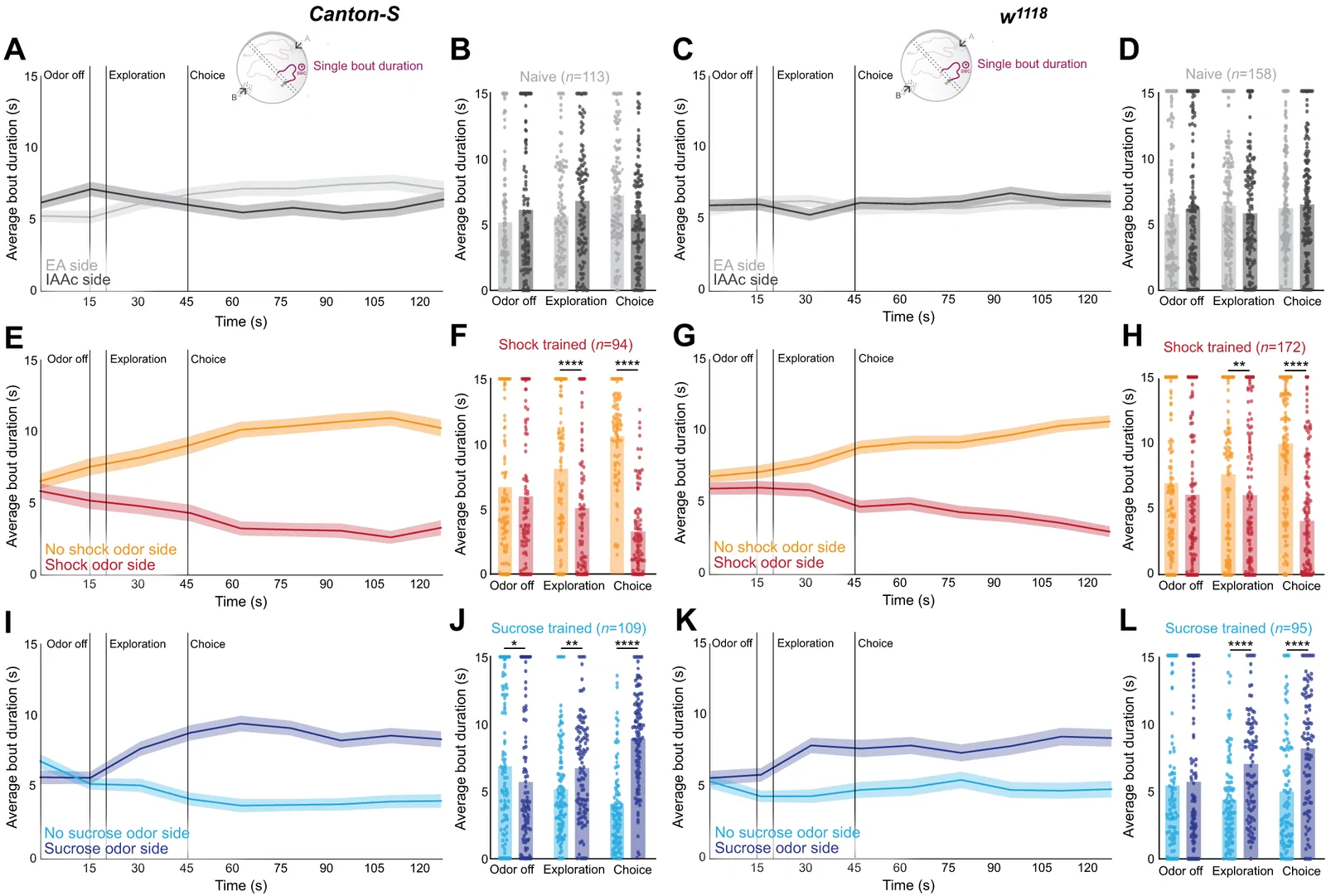
**Bout duration reflects memory.** Bout duration on each arena side was binned every 15 s, reflecting an average transition rate of less than one every 10 s in all groups (Fig. S12). Line graphs depict mean±s.e.m. bout duration per 15 s bin and bar graphs depict mean bout duration per bin in the choice phase, where each dot represents the mean bout duration per bin of a single fly. Neither naive *Canton-S* (A,B) or *w^1118^* (C,D) flies waited longer to transition from either side over time. Shock-trained *Canton-S* (E,F) and *w^1118^* (G,H) flies waited longer to transition out of the odor without shock pairing over time. Sucrose-trained *Canton-S* (I,J) and *w^1118^* (K,L) flies waited longer to transition out of the sucrose-paired odor over time. Asterisks denote significant differences (*P<0.05, **P<0.01, ****P<0.0001). Samples, statistical tests and detailed results are given in Table S2. Experimental replicate and fly exclusion details are given in Table S4.

These results indicate that during the exploration and choice phases, flies continued to transition between odors but with increasingly longer bouts in the side they preferred. Over time, they more rapidly exited the odor previously paired with shock and lingered with the odor previously paired with sucrose. Together with regional distance traveled, mean bout duration may reflect the choice between different stimuli in associative memory assays.

### Behaviors that reflect that a decision has been made

We next sought to identify metrics within the choice phase that indicate the maintenance of a decision (Fig. 4). We first evaluated midline U-turns, instances where flies approached the center of the arena before turning back to remain on the same side (Fig. 4A; for parameters, see Fig. S6). As the number of U-turns depended on locomotor activity, the metric was normalized as average U-turns per cm traveled. Naive *Canton-S* flies did not make more U-turns on either side (Fig. 4B, *P*=0.16), but shock-trained *Canton-S* flies made fewer U-turns on the shock-paired odor side (Fig. 4C, *P<*0.0001), and sucrose-trained *Canton-S* flies made more U-turns on the sucrose-paired odor side (Fig. 4D, *P*=0.03). Similar to *Canton-S* flies, naive *w^1118^* flies did not make more U-turns (Fig. 4E, *P*=0.32). However, unlike *Canton-S* flies, shock-trained *w^1118^* flies did not make more U-turns per cm (Fig. 4F, *P*=0.16), perhaps because of a greater reduction in overall locomotion than shock-trained *Canton-S* flies (Fig. S11C,E). Sucrose-trained *w^1118^* flies made more U-turns on the sucrose-paired odor side (Fig. 4G, *P*=0.003). Together, these results suggest that once flies have decided between stimuli, they actively reject the alternative stimulus.

**Fig. 4.**
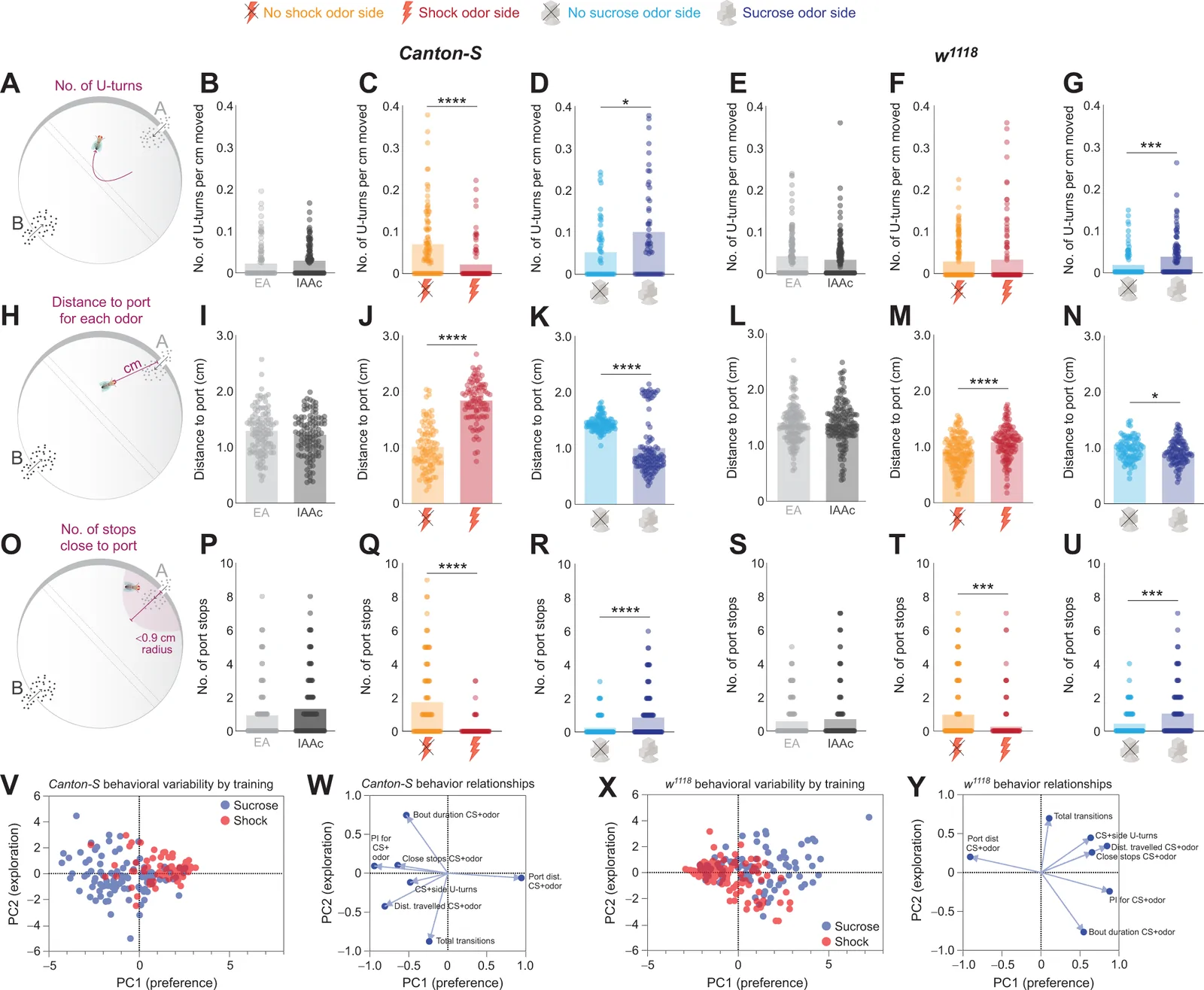
**Behaviors reflect memory-based decisions.** Bar graphs depict means across flies and dots depict values of individual flies. (A) Graphic depiction of a U-turn. (B–G) Naive flies did not make more U-turns on either side, while trained flies made more U-turns when occupying the arena side containing the preferred odor stimulus (sucrose paired or no-shock paired), except for shock-trained *w^1118^* flies. (H) Graphic depiction of the distance to an odor port on a given arena side. (I–N) Naive flies did not stay closer to either port, but trained flies stayed closer to the port emitting the preferred odor. (O) Graphic depiction of a stop close to an odor port. (P–U) Naive flies did not make more stops close to either odor port, but trained flies made more stops close to the preferred odor stimulus. (V,X) Principal component scores of individual *Canton-S* (V) and *w^1118^* (X) flies depict behavioral variability between sucrose- and shock-trained groups. (W,Y) Loadings depict how individual behaviors of *Canton-S* (W) and *w^1118^* (Y) flies group to form the principal components of odor preference (PC1) and exploration (PC2). Each behavior included in the principal component analysis (PCA) showed a memory-dependent difference between arena sides (B–U). When occupying the side containing the conditioned odor stimulus (CS+), behaviors that correlated with a learned preference included the average distance from the source of the odor (odor port distance), the number of stops made close to the CS+ odor port, and the number of U-turns away from the alternative odor. Distance traveled on the CS+ side correlated more with preference in *w^1118^* flies than in *Canton-S* flies. Behaviors that correlated least with preference were the total number of arena side transitions made and average bout duration on the CS+ odor side, with greater transitions and lower average bout durations reflecting a less clear odor preference. Asterisks denote significant differences (*P<0.05, ***P<0.001, ****P<0.0001). Samples, statistical tests and detailed results are given in Table S2. Experimental replicate and fly exclusion details are given in Table S4.

The average proximity of flies from each odor port while occupying each arena side was also calculated during the choice phase (Fig. 4H). We hypothesized that, when occupying their respective arena sides, flies would stay closer to the preferred stimulus than to the other stimulus. Naive *Canton-S* flies did not get closer to ports emitting either odor (Fig. 4I, *P*=0.13), but shock-trained *Canton-S* flies stayed farther from the port emitting the shock-paired odor (Fig. 4J, *P<*0.0001), and sucrose-trained *Canton-S* flies stayed closer to the port emitting the sucrose-paired odor (Fig. 4K, *P<*0.0001). Similarly, naive *w^1118^* flies also did not get closer to either odor port (Fig. 4L, *P*=0.57), whereas shock-trained *w^1118^* flies stayed farther from the port the emitting shock-paired odor (Fig. 4M, *P<*0.0001) and sucrose-trained *w^1118^* flies stayed closer to the port emitting the sucrose-paired odor (Fig. 4N, *P*=0.04).

Given that flies stayed closer to their preferred stimulus in the choice phase, we hypothesized that they would also stop more frequently close to the port emitting the preferred odor during the choice phase. Stops were defined as specified previously (Branson et al., 2009), and proximity to the odor port was defined as being within 0.9 cm of the port (Fig. 4O). Naive *Canton-S* flies showed no difference in the total number of close-to-port stops on either side (Fig. 4P, *P*=0.052), while shock-trained *Canton-S* flies made fewer stops close to the shock-paired odor port (Fig. 4Q, *P<*0.0001) and sucrose-trained *Canton-S* flies made more stops close to the sucrose-paired odor port (Fig. 4R, *P<*0.0001). Similarly, naive *w^1118^* flies did not make more stops close to either port (Fig. 4S, *P*=0.90), while shock-trained *w^1118^* flies made fewer stops close to the shock-paired odor port (Fig. 4T, *P*=0.0002) and sucrose-trained *w^1118^* flies made more stops close to the sucrose-paired odor port (Fig. 4U, *P*=0.0002).

To understand which behavior metrics best represent shock or sucrose memory, we performed PCA (Fig. 4V,X). Principal component 1 (PC1) described odor preference by best correlating with PI and mean odor port distance, and accounted for 49.01% of behavioral variance in *Canton-S* flies and 49.51% of variance in *w^1118^* flies. PC2 described exploration between odors by correlating with transitions between arena sides and average bout duration (Fig. 4W,Y) and accounted for 22.02% of variance in *Canton-S* flies and 22.13% of variance in *w^1118^* flies*.* Together, PC1 and PC2 explained ∼71% of behavioral variance in both *Canton-S* and *w^1118^* flies, suggesting that the measured behaviors with the greatest change in response to associative olfactory learning were those that reflected odor preference. Unsurprisingly then, the greatest differences between shock- and sucrose-trained groups were their opposite preference for the conditioned odor (paired with shock versus sucrose) and their average distance from the respective odor ports. Shock-trained flies also appeared to behave more uniformly than sucrose-trained flies (Fig. 4V,X), and when occupying the side with shock-paired odor, their choice may be primarily characterized by a farther distance from the odor port. Conversely, greater average U-turns, distance traveled and close stops to the conditioned stimulus odor port may have co-occurred in sucrose-trained flies that preferred the conditioned odor. The evaluation of U-turns, distance to port and number of stops close to port collectively suggests that flies exhibited discrete behaviors when navigating between two conditioned odor stimuli that reflect their choice of preferred odor.

### *dunce* mutants exhibit reduced learning and choice-related behaviors in the assay

To test whether the two-choice assay was effective for identifying mutations known to reduce memory, we tested whether *dunce*, a gene that encodes a cAMP phosphodiesterase, impacts learned odor choice (Dudai et al., 1976; Byers et al., 1981; Qiu and Davis, 1993). *dunce* mutants express a truncated version of cAMP phosphodiesterase that does not inactivate cAMP, leading to cAMP overexpression that fails to regulate synaptic plasticity (Byers et al., 1981; Davis and Kiger, 1981; Davis, 1996; Gervasi et al., 2010). As a result, *dunce* mutants exhibit reduced appetitive and aversive learning and memory (Dudai et al., 1976; Tempel et al., 1983).

We assessed whether *dunce* reduced preference for an odor associated with either shock or sucrose compared with its genetic control (*Canton-S*) by recording PI and analyzing post-choice behaviors (Fig. 5). Additionally, individual PI was compared between the odor-off and choice phases for each genotype to assess within-subject memory (Fig. S13). As expected, *Canton-S* controls demonstrated both shock memory (Fig. S13A, *P<*0.0001) and sucrose memory (Fig. S13B, *P*=0.009). While *dunce* mutants exhibited memory of the shock-paired odor (Fig. S13C, *P*=0.004), their memory was significantly reduced compared with that of *Canton-S* flies (Fig. 5A,C, *P<*0.0001). *dunce* mutants did not demonstrate significant sucrose memory (Fig. S13D, *P*=0.20), which also resulted in decreased preference for sucrose-paired odors compared with *Canton-S* controls (Fig. 5B,G, *P*=0.001).

**Fig. 5.**
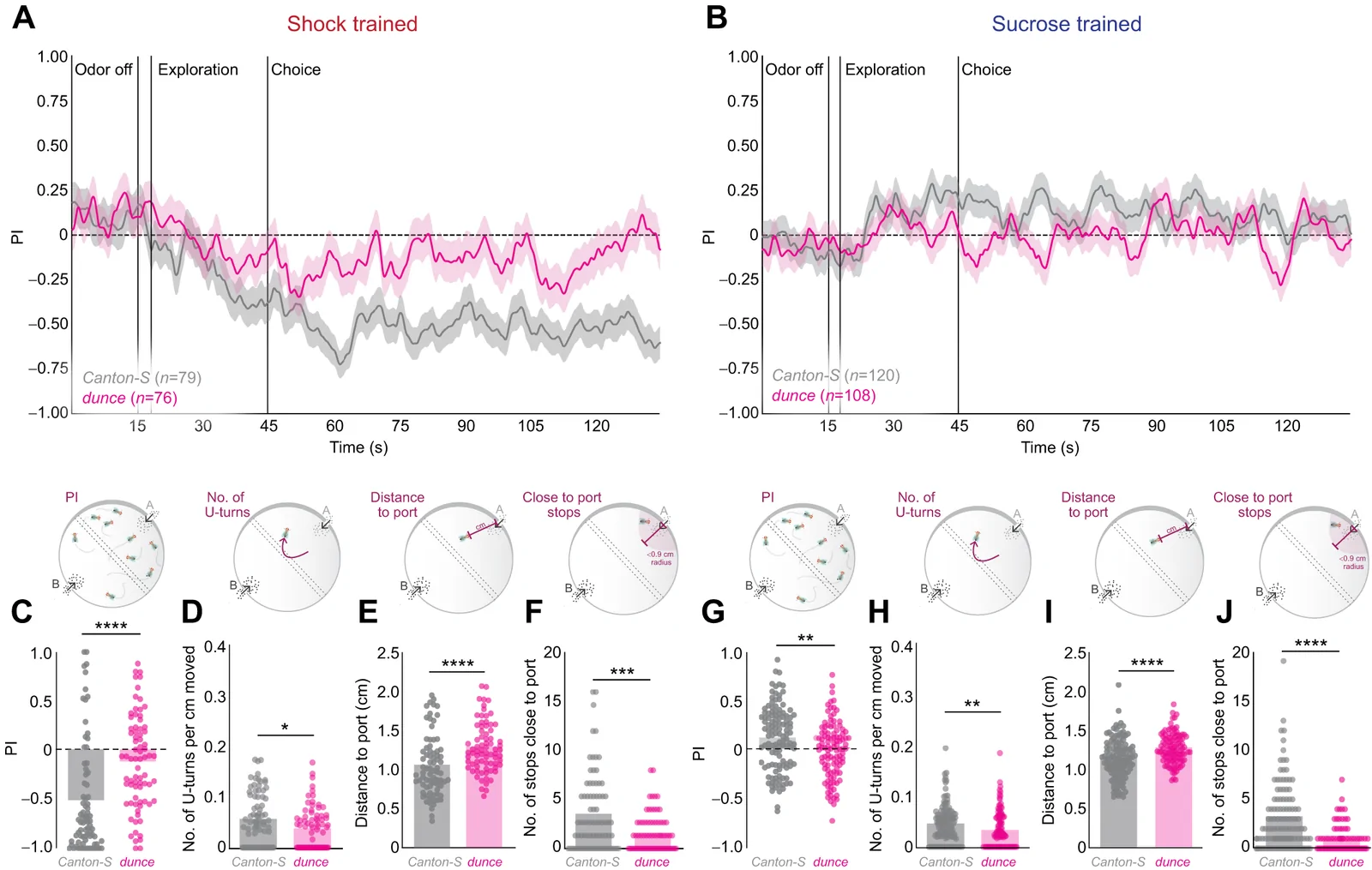
***dunce* mutants exhibit blunted learning and post-choice behaviors.** PI and behaviors in the choice phase were compared between wild-type *Canton-S* flies and *dunce* mutants. Line graphs depict mean±s.e.m group PI. Bar graphs depict means with dots representing values of individual flies. (A,B) Average group PI over time for the shock-paired (A) and sucrose-paired (B) odors by each genotype. (C–F) Shock-trained *Canton-S* flies exhibited greater individual preference for the odor without shock pairing (C) and more robust choice behavior by making fewer U-turns on the shock-paired odor side (D), staying farther away from the shock-paired odor (E) and making fewer stops close to the shock-paired odor port (F) than shock-trained *dunce* flies. (G–J) Sucrose-trained *Canton-S* flies exhibited greater individual preference for the sucrose-paired odor (G) and more robust choice behavior by making more U-turns on the sucrose-paired odor side (H), staying closer to the source of the sucrose-paired odor (I) and more frequently stopping close to the sucrose-paired odor port (J) than sucrose-trained *dunce* flies. Asterisks denote significant differences (*P<0.05, **P<0.01, ***P<0.001, ****P<0.0001). Samples, statistical tests and detailed results are given in Table S2. Experimental replicate and fly exclusion details are given in Table S4.

We next tested whether the strength of choice behaviors was greater in *Canton-S* flies than in *dunce* flies. Shock-trained *Canton-S* flies made more U-turns on the side containing odor without shock pairing (Fig. 5D, *P*=0.02), remained closer to the no-shock paired odor port (Fig. 5E, *P<*0.0001), and made more stops close to the no-shock paired port (Fig. 5F, *P<*0.0001) compared with shock-trained *dunce* flies. Sucrose-trained *Canton-S* flies also made more U-turns per cm traveled on the side containing the sucrose-paired odor (Fig. 5H, *P*=0.002), remained closer to the port emitting the sucrose-paired odor (Fig. 5I, *P<*0.0001), and made more stops close to the port emitting the sucrose-paired odor (Fig. 5J, *P<*0.0001) than their *dunce* counterparts. These metrics indicate that the *dunce* mutation significantly decreased memory expression through a lack of robust choice behavior, suggesting the two-choice assay and identified choice behaviors are useful for identifying genes and circuits that impact memory formation.

## DISCUSSION

We introduce a high-throughput olfactory two-choice assay to test associative memory that captures individual and group behavior over time to better characterize behavioral choice. Using the assay, we show that conditioned flies exhibit a sustained collective preference between odor stimuli within 30 s of odor introduction. This interval mirrors the speed of decision making previously reported in shock-trained flies (Ichinose and Tanimoto, 2016) and suggests that choice occurs in the same time frame following appetitive conditioning. Furthermore, we found that flies exhibit distinct locomotor dynamics between each odor stimulus that reflect their decision. The largest differences between stimuli typically occurred following the first 30 s of odor exposure (during the choice phase), coinciding with the latency for collective decision making.

Within the choice phase, we observed additional behaviors that indicate active avoidance of an odor previously paired with an aversive stimulus, and engagement with an odor previously paired with a rewarding stimulus, including the length of bouts near the paired odor, the number of U-turns toward the paired odor, proximity to the odor port and the number of stops near the odor port. Specifically, in the presence of the conditioned odor, shock training was represented by a farther distance from the paired odor, while sucrose training was represented by more U-turns toward the paired odor and closer stops to the paired odor port. Given our findings of greater locomotor activity on the preferred odor side, the closer proximity and more frequent stops near the preferred port suggest that sucrose-trained flies may have engaged in foraging or seeking behavior near the sucrose-paired odor, while the shock-trained flies may have attempted to get as far as possible from the shock-trained odor source. Thus, rather than a single static measurement, our results reflect decision making as a series of behavioral strategies exhibited across stimulus exposure.

Our data also revealed that trained flies continuously transitioned between arena sides throughout the experiment, although the overall number of transitions decreased in shock-trained flies (Fig. S12). This suggests that even after a collective choice was made, individual flies did not strictly limit their exposure to the preferred stimulus. Indeed, one study showed that flies' expression of olfactory memory depends on access to comparison between the two odors during testing (Modi et al., 2023), suggesting that by transitioning between odors over the course of the experiment, flies engaged in comparison between the odors in the process of remembering. We additionally found that over time, both sucrose- and shock-trained flies reduced their bout time on the side with an odor they wanted to avoid and increased their bout time on the side with an odor they preferred, suggesting that comparison between odors decreased but did not stop once a collective choice was made.

Our findings thus add to the growing literature that flies, like humans, exhibit individual variability in ecologically relevant behaviors such as memory performance (Werkhoven et al., 2021; De Bivort et al., 2022; Smith et al., 2022; Godesberg et al., 2024). This variation could be driven by different individual experiences during either odor–shock or odor–sucrose training, or in memory expression. While individual flies show similar short-term olfactory memory performance to flies trained and tested as a group (Platt et al., 1980; Tempel et al., 1983; Phelan et al., 1998), social interactions during testing may alternatively facilitate anesthesia-resistant memory (Chabaud et al., 2009). The ability to observe individual flies behaving within a group with immediate access to both odor stimuli suggests the two-choice assay provides an advantageous platform for future studies seeking to further understand how group behavior influences differing memory types. To this end, an important future development includes monitoring behavior during training sessions within arenas similar to the two-choice to study how individuals and groups interact with the unconditioned stimuli.

Taken together, our findings show that flies exhibit a range of behaviors to make a choice between stimuli that parallels findings in rodent (Izquierdo and Belcher, 2012; Ahmed, 2018; Roy et al., 2021) and human studies (Camchong et al., 2014; Cabeza et al., 2020; Midroit et al., 2021). Behaviorally distinct pre- and post-choice time intervals (here called exploration and choice phases) phenocopy those demonstrated in rats and humans (Fam et al., 2016). Additionally, U-turns mirror vicarious trial and error, namely the act of turning between stimulus options in rats (Redish, 2016), while stops close to odor ports appear to recapitulate findings of decreased velocity near food-paired odors in mice (Liu et al., 2020). As a model organism with high genetic tractability (Venken et al., 2011) and unique resources such as the whole-brain connectome (Zheng et al., 2018; Dorkenwald et al., 2024), increasing the scope of behavioral analysis in *D. melanogaster* has the potential to accelerate efforts to understand general neurobiological principles underlying decision making. We demonstrated that this simple two-choice behavioral tracking assay effectively identifies mutations that impact memory and provides a sensitive high-throughput method for capturing the dynamic process of memory-based choice with enhanced resolution.

## Supplementary Material

10.1242/jexbio.253164_sup1 Supplementary information

Table S2. Main Figure Statistics and N counts.

Table S3. Supplemental figure statistics and N counts.

Table S4. Fly and frame exclusions per group.
