# Supplementary material for: Characterizing choice behavior in Drosophila melanogaster olfactory classical conditioning
Source: J Exp Biol. 2026 Oct 13;229(19):jeb253164. doi: 10.1242/jeb.253164 (PMC13723394; doi:10.1242/jeb.253164)
Supplement: Supplementary file 1 [file jexbio-229-253164-s1.pdf]

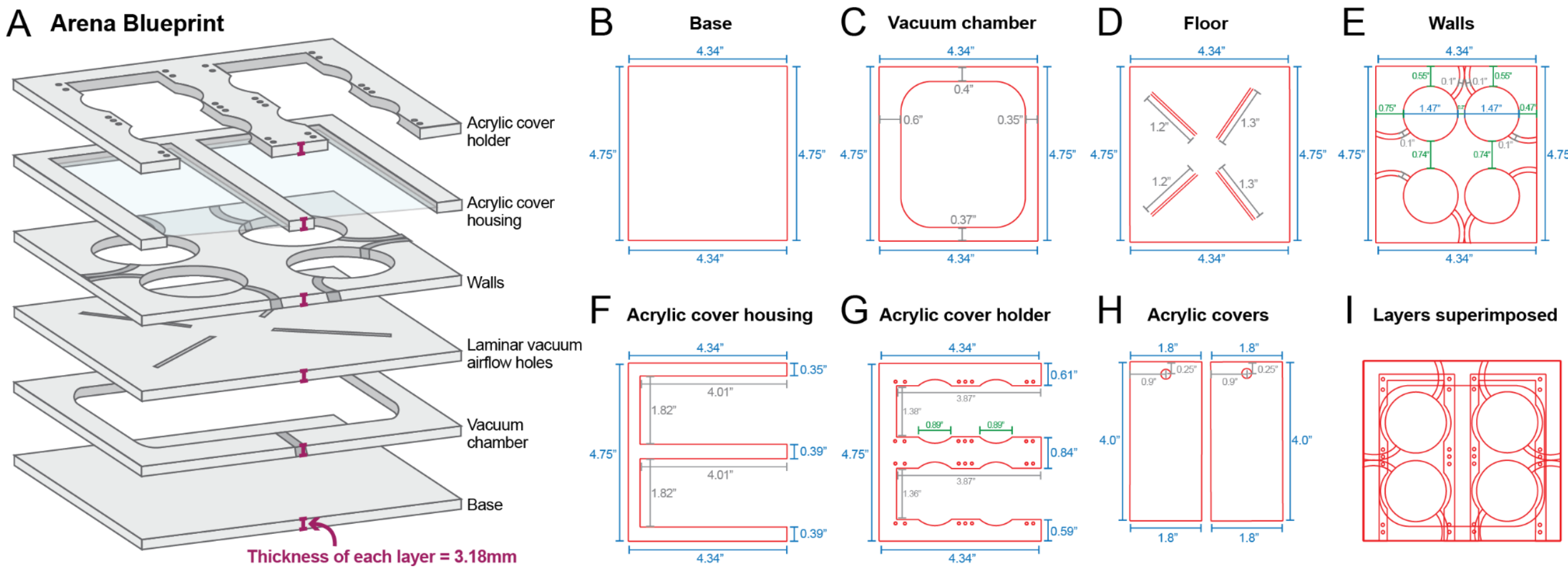

Fig. S1. Layered diagram of the Two-Choice apparatus.

(A) The two-choice apparatus consists of six separately laser-cut Teflon sheets sealed with plastic adhesive (Loctite, 681925, Rocky Hill, Connecticut). The bottom two layers **(B-C)** form a pocket of air sucked out of the arenas by the vacuum tube attached to the vacuum chamber layer. The middle two layers **(D-E)** form the floor and walls of the four arenas featured in the assay. The top two layers **(F-G)** contain the housing for the removable plexiglass arena covers **(H)** and screw holes to fasten the covers in place, forming the superimposed apparatus **(I)**.

Journal of Experimental Biology • Supplementary information

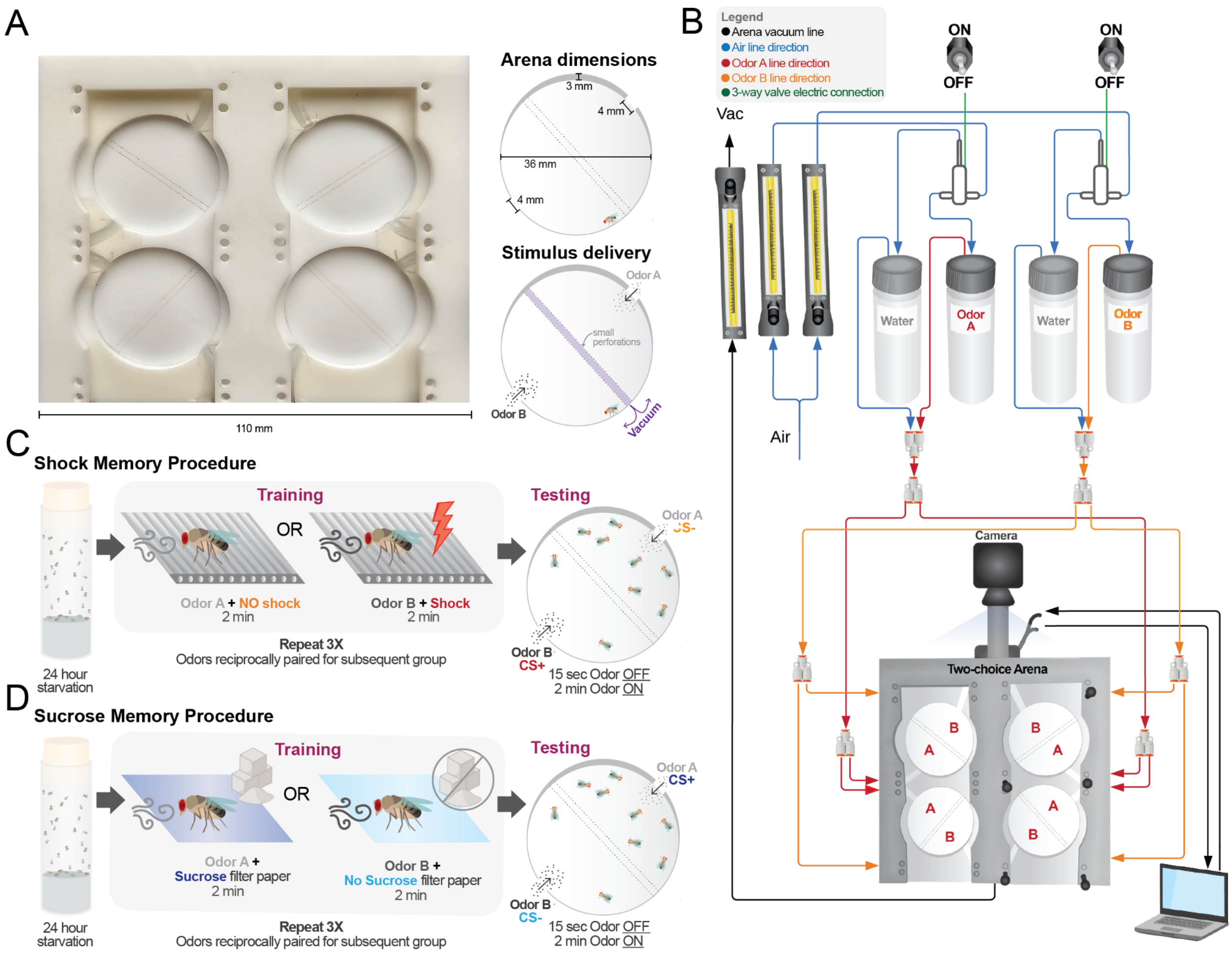

Fig. S2. Two-Choice setup and experimental procedures (A)
The behavioral arenas from above (left), along with arena dimensions (top right) and features for stimulus delivery (bottom right). **(B)** Odor delivery setup. Air and vacuum flow rates are adjustable by separate flowmeters. Airflow can be switched from a 3-way valve to pass through either an odor or water vial before reaching the arenas. After passing through a vial, air is forked into two tubes to reach two arenas at once. Forked tubes can be detached and reconnected to switch the odor emitted at each arena side between trials. **(C-D)** Graphic of shock and sucrose memory experimental procedures. Food-deprived flies were trained to associate an odor (ethyl acetate or isoamyl alcohol) with 80V electric shock or 2M sucrose. Flies were initially exposed to an odor without sucrose or shock for two minutes, then exposed to the other odor paired with sucrose or shock over the following two minutes. This training cycle was repeated a total of three times. Immediately after training, 10 flies per arena were aspirated into the apparatus and exposed to both odors for two minutes to test odor preference.

Journal of Experimental Biology • Supplementary information

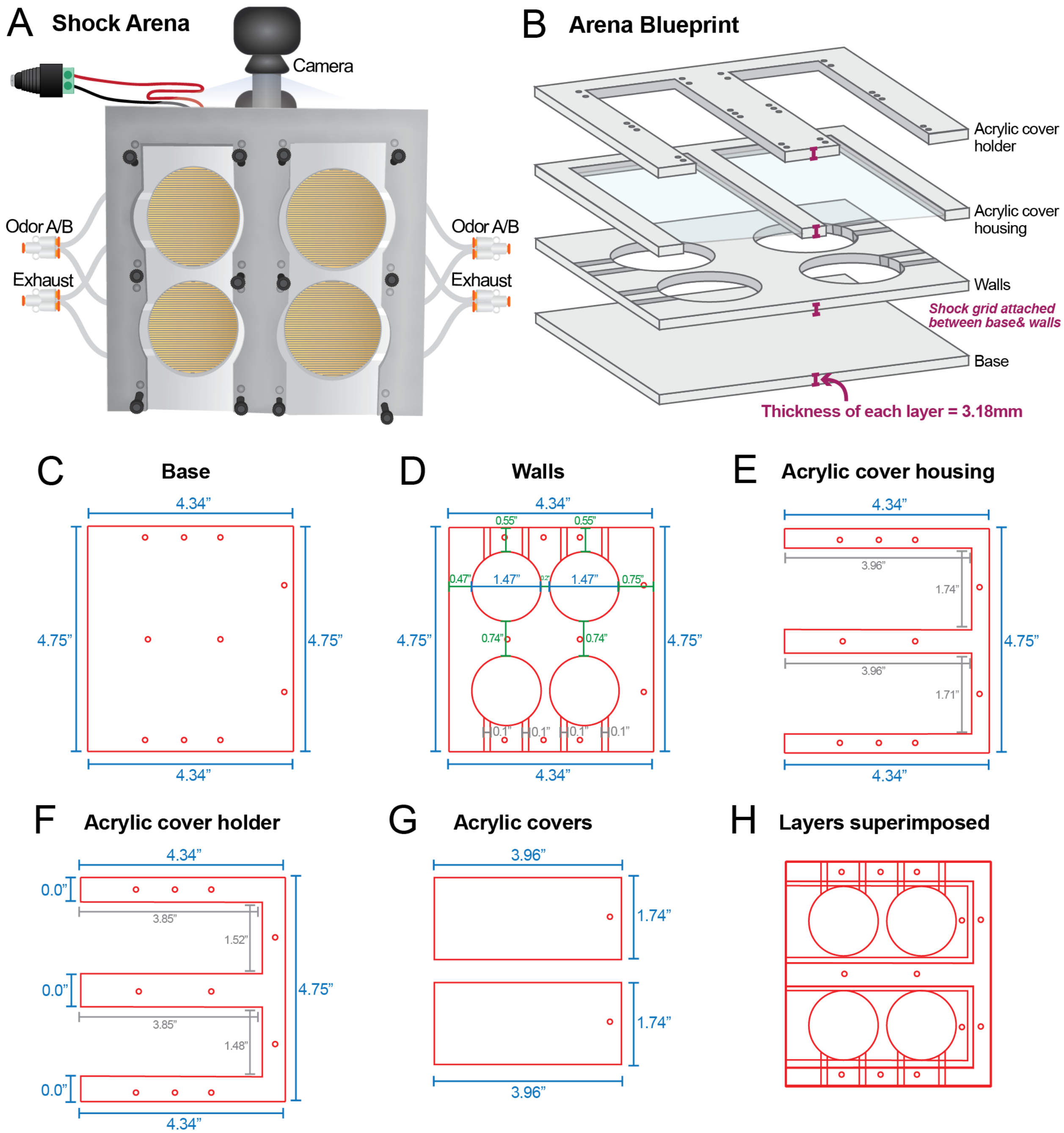

Fig. S3. Shock training arena.

(A) Illustration of shock arena used during training, adopted from (Scaplen *et al.*, 2019). Herein, odors flow one at a time through single tubes to each arena. Each electrode surface beneath the copper grating is connected individually to hookup wires fed into a single connector that can be plugged into the pulse stimulator. **(B)** Layard diagram of the shock arena. **(C-G)** Individual layer dimensions. **(H)** Final Behavioral arena dimensions match the two-choice assay.

Journal of Experimental Biology • Supplementary information

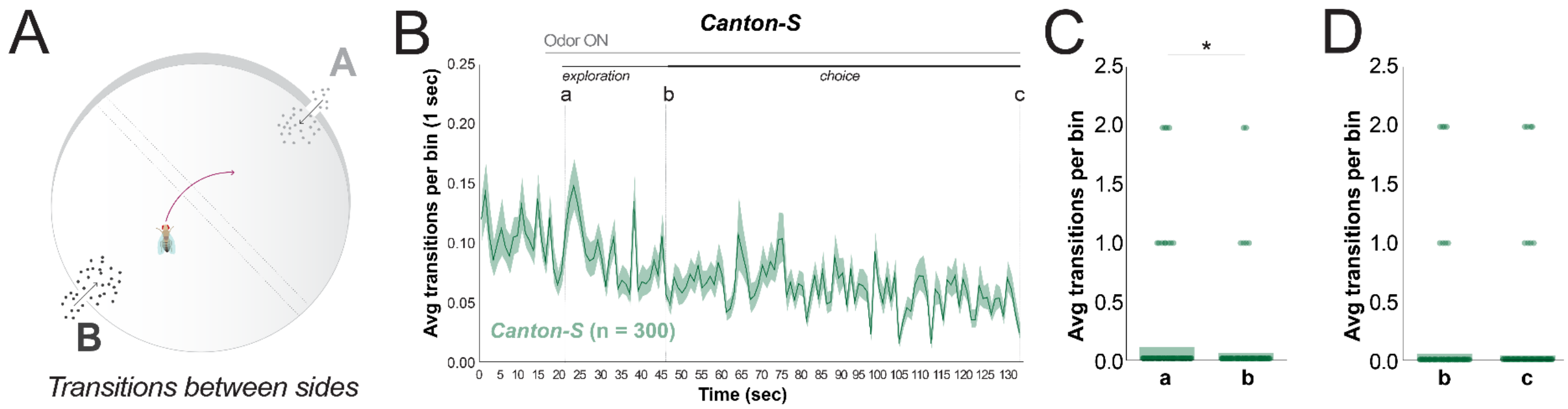

Fig. S4. *Canton-S* 1-second bin transitions over time

(A) Graphic of a transition between arena sides. **(B)** mean ± s.e.m transitions per-second of naïve *Canton-S*. **(C)** When average transitions were binned per-second, *Canton-S* flies made fewer transitions in the bin that occurred 30 seconds after odor introduction (bin B) compared to the bin that occurred 5 seconds after odor introduction (bin A) (p = 0.02). **(D)** *Canton-S* did not make fewer transitions at 90 seconds after odor introduction (bin C) compared to 30 seconds after odor introduction (bin B) (p = 0.41). Samples, statistical tests and detailed results are found in the S.Figure 4 subsection of Table S3. Experimental replicate and fly exclusion details are found in Table S4.

Journal of Experimental Biology • Supplementary information

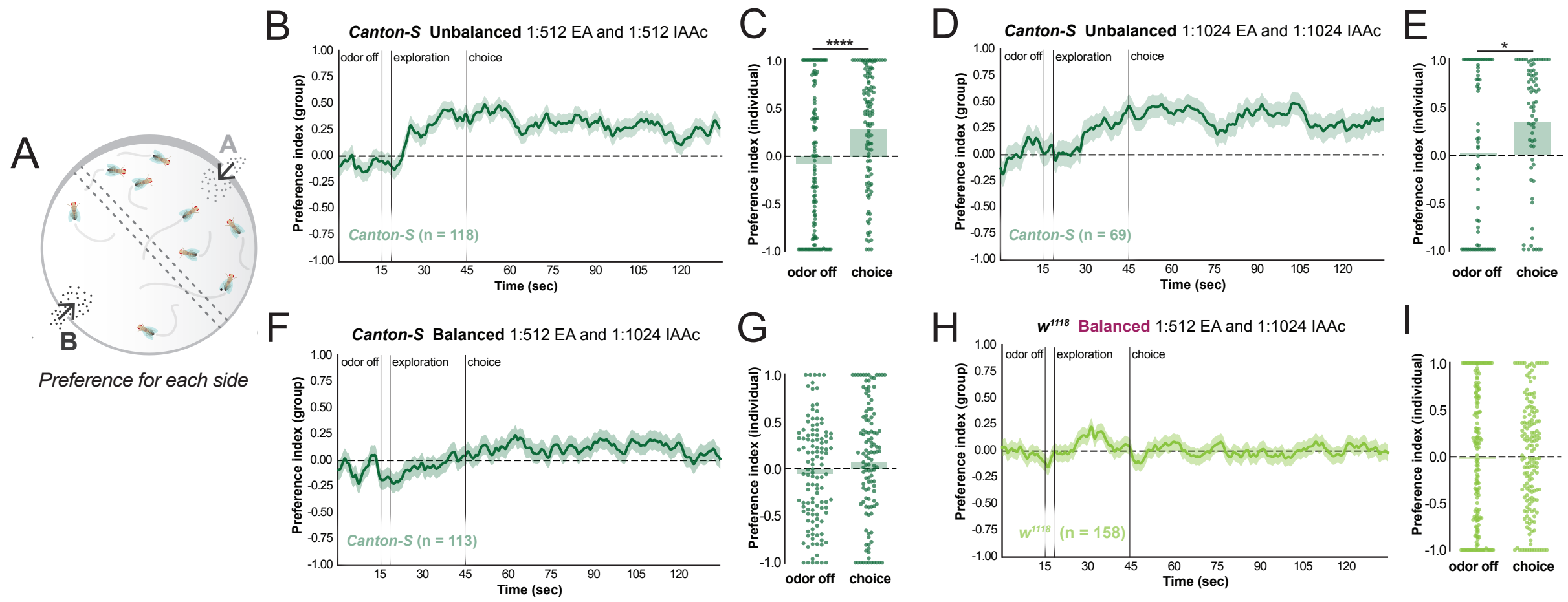

Fig. S5. Preference for varying odor concentrations.

Naïve flies were exposed to differing concentrations of EA and IAAc following starvation to find a pair of concentrations that did not evoke an odor bias. Line graphs depict mean ± s.e.m of group preference index and bar graphs depict mean individual preference index, with dots representing individual values per fly. **(A)** Graphic of per-group preference index as the distribution of flies across each arena side over time. **(B-C)** Naïve *Canton-S* flies show a group and individual preference for 1:512 EA over 1:512 IAAc after 25 seconds of odor exposure ($p < 0.0001$). **(D-E)** Naïve *Canton-S* flies show a group and individual preference for 1:1024 EA over 1:1024 IAAc within 25 seconds of odor exposure ($p = 0.02$). Neither Naïve *Canton-S* **(F-G)** or w^{1118} **(H-I)** flies show a group or individual preference for 1:512 EA over 1:1024 IAAc after 25 seconds of odor exposure (*Canton-S* $p = 0.33$, w^{1118} $p = 0.8$). Samples, statistical tests and detailed results are found in the S.Figure 5 subsection of Table S3. Experimental replicate and fly exclusion details are found in Table S4.

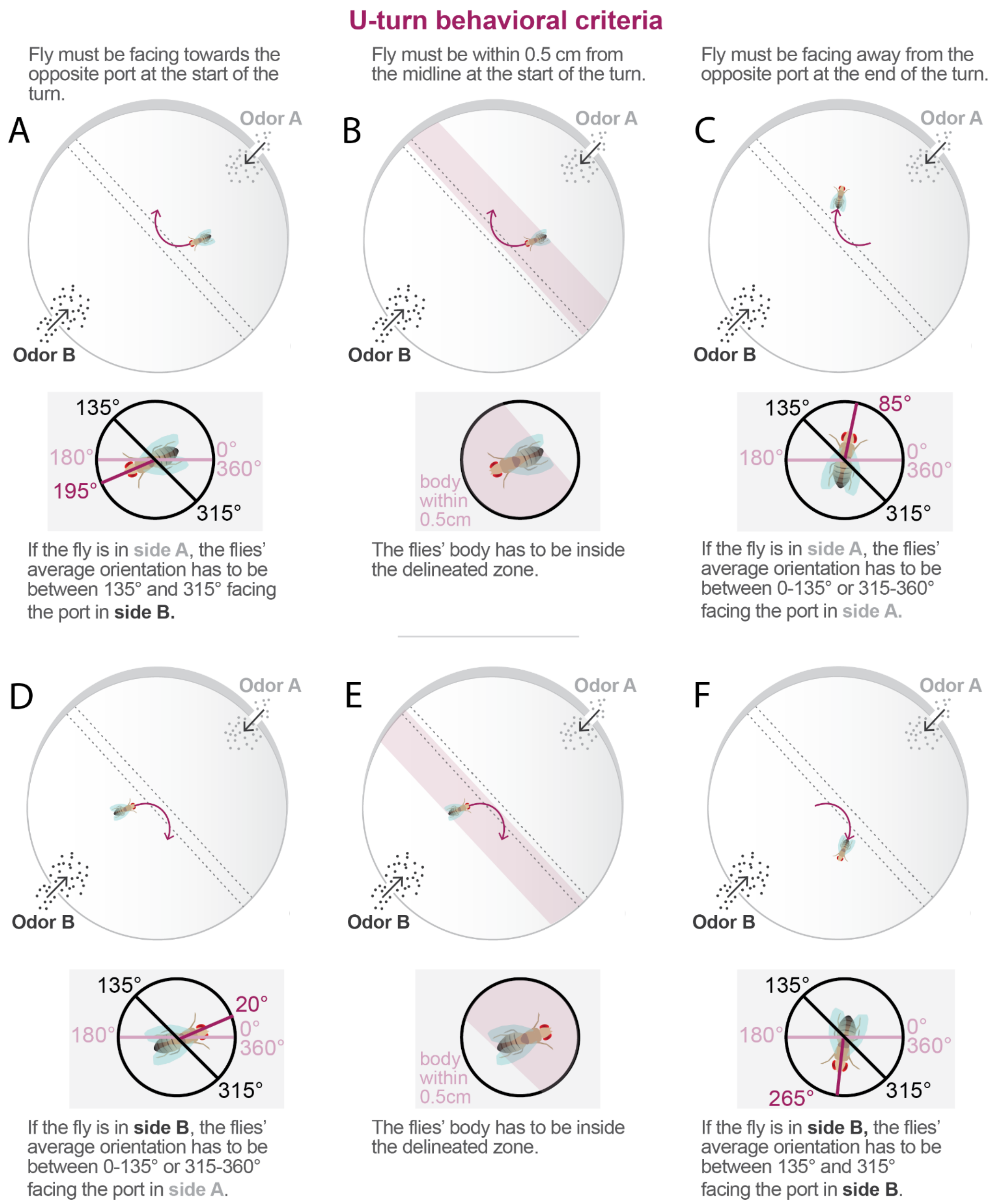

Fig. S6. U-turn away from midline parameters on each arena side.

An example arena where the midline is at a 135° angle from the x axis is illustrated. A U-turn must first meet the per-frame criteria of a sharp turn as delineated by (Branson *et al.*, 2009) for at least five frames. In order for a fly on the top arena side to make a U-turn, it must **(A)** be oriented towards the midline (135°-315°) in the five frames preceding the start of the turn, **(B)** start the turn within 0.5 cm of the midline, and **(C)** be oriented away from the midline (0°-135°)U(315°-360°) on the side the turn was initiated for at least five frames. In order for a fly on the bottom arena side to make a U-turn, it must **(D)** be oriented towards the midline (0°-135°)U(315°-360°) in the five frames preceding the start of the turn, **(E)** start the turn within 0.5 cm of the midline, and **(F)** be oriented away from the midline (135°-315°) on the side the turn was initiated for at least five frames.

Journal of Experimental Biology • Supplementary information

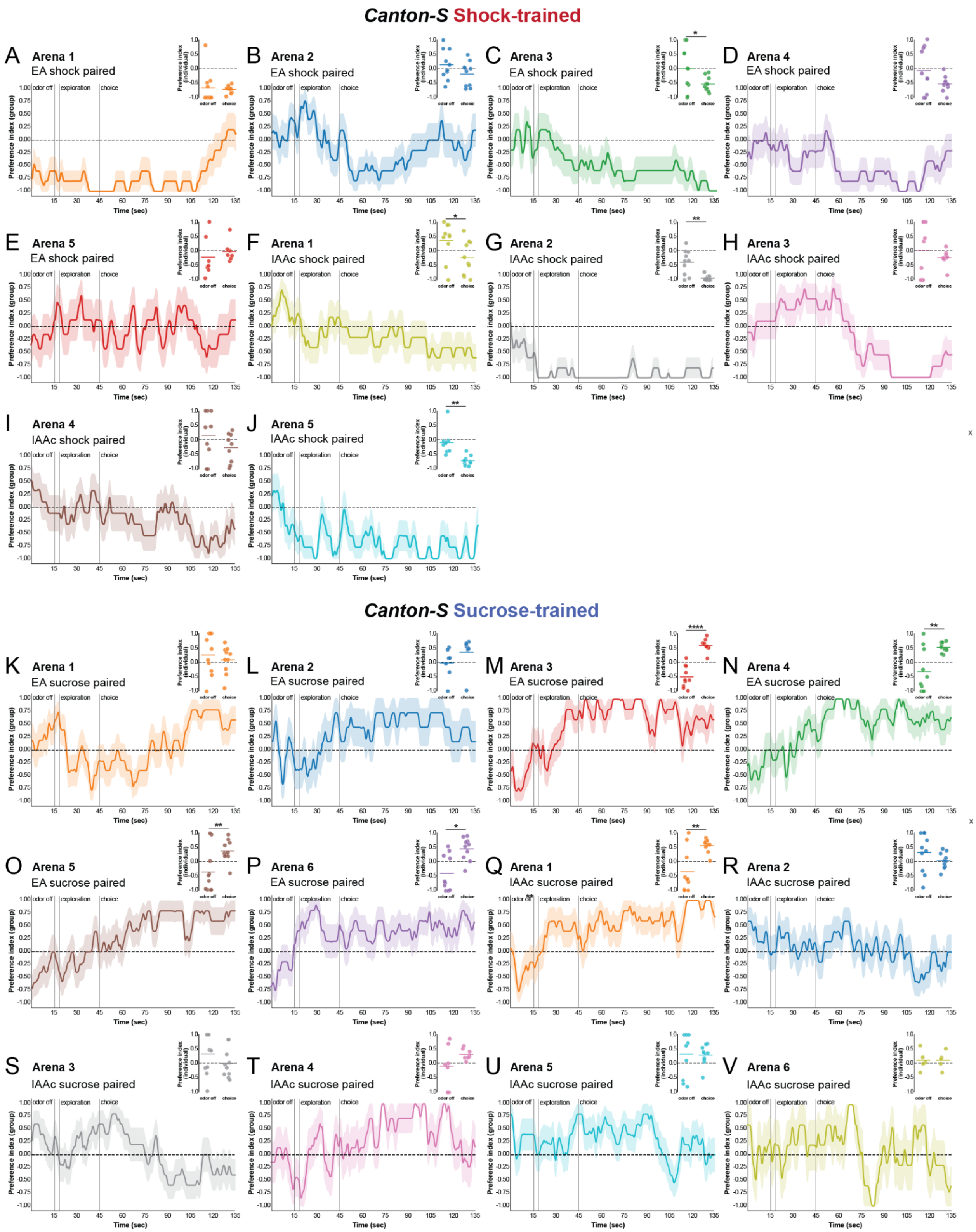

Fig. S7. Conditioned *Canton-S* preference index per arena.

Ten flies were tested per arena. Line graphs depict mean ± s.e.m group preference index across flies in one arena over time. Bar graphs depict the mean individual preference index of each fly averaged across frames during the odor-off and choice phases as a

dot, and the mean across flies as a bar. Positive preference index indicates preference for the conditioned stimuli (odor paired with shock or sucrose). (A-J) In the majority of arenas, shock-trained flies show a tendency to avoid the odor paired with shock. (K-V) In the majority of arenas, sucrose-trained flies show a tendency to approach the odor paired with sucrose. Samples, statistical tests and detailed results are found in the S.Figure 7 subsection of Table S3. Experimental replicate and fly exclusion details are found in Table S4.

Journal of Experimental Biology • Supplementary information

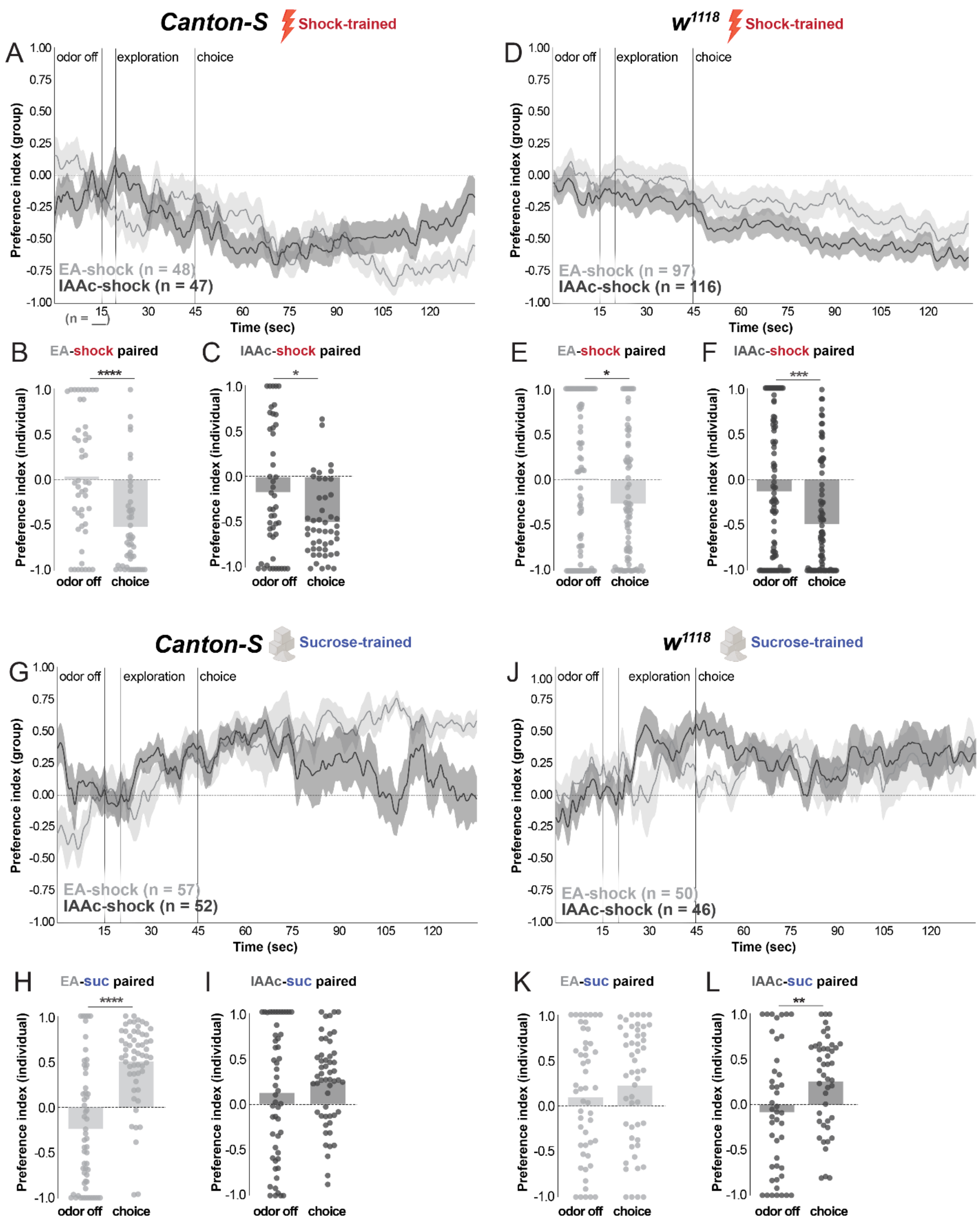

Fig. S8. Strength of exhibited associative learning depends on odor.

Line graphs depict mean ± s.e.m of group preference index. Bar graphs depict mean individual preference index, with dots representing individual values per fly. **(A)** Shock-trained *Canton-S* average group preference index over time grouped by shock-paired odor. **(B-C)** Effect size of individual preference index was greater *Canton-S* that received shock paired with EA than in those that received shock paired with IAAc; (EA $p < 0.0001$, IAAc $p = 0.02$). **(D)** Shock-trained w^{1118} average group preference index over time grouped by shock-paired odor. **(E-F)** Effect size of individual preference index was greater in w^{1118} flies that received shock paired with IAAC than in those that received shock paired with EA (EA $p = 0.02$, IAAc $p = 0.0004$). **(G)** Sucrose-trained *Canton-S* average group preference index over time grouped by sucrose-paired odor. **(H-I)** Sucrose pairing with EA but not IAAc resulted in associative memory in *Canton-S* flies (EA $p < 0.0001$, IAAc $p = 0.2$). **(J)** Sucrose-trained w^{1118} average group preference index over time

Journal of Experimental Biology • Supplementary information

grouped by sucrose-paired odor. **(K-L)** Sucrose pairing with IAAc but not EA resulted in associative memory in w^{1118} flies (EA p = 0.18, IAAc p = 0.004). Samples, statistical tests and detailed results are found in the S.Figure 8 subsection of Table S3. Experimental replicate and fly exclusion details are found in Table S4.

Journal of Experimental Biology • Supplementary information

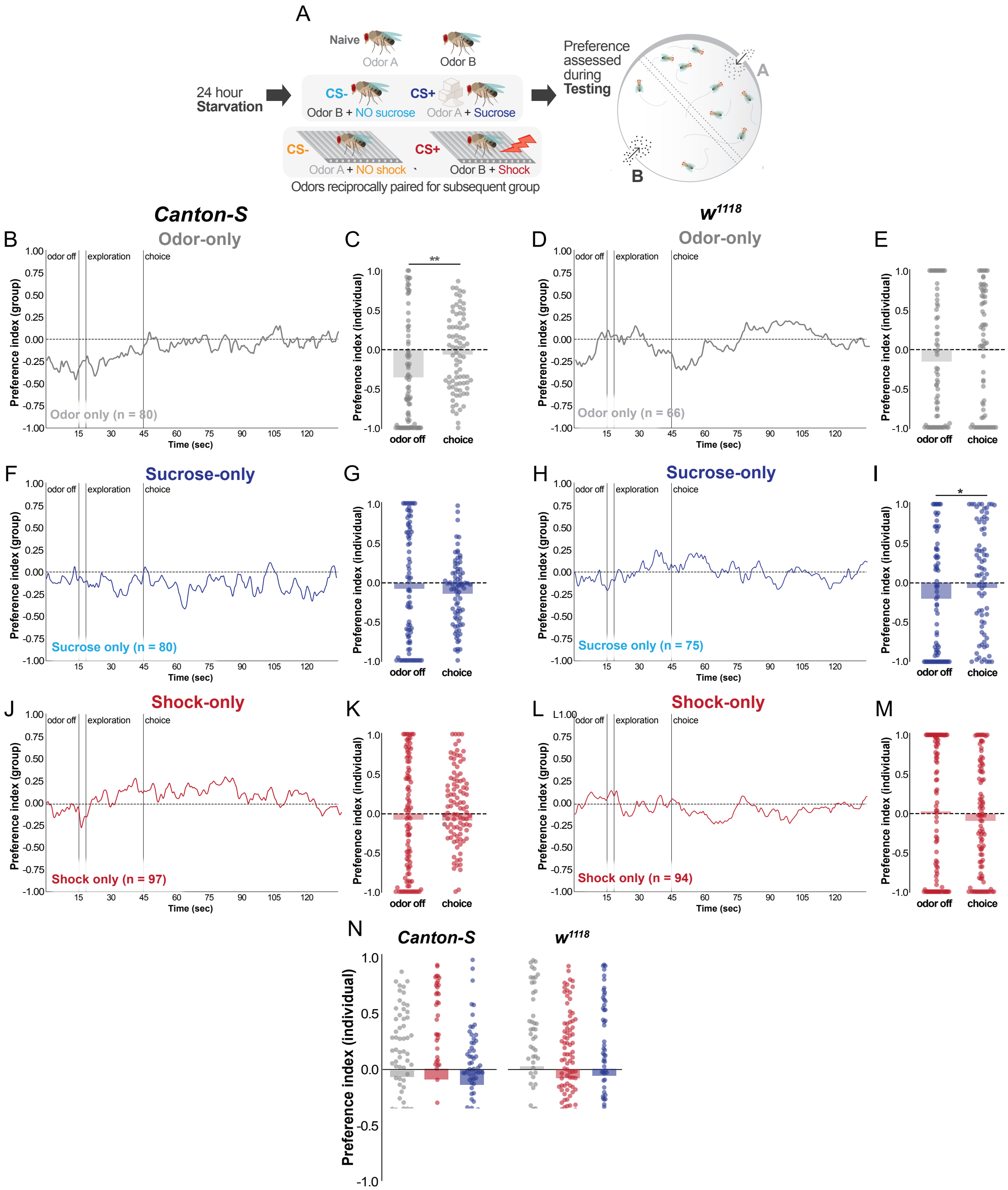

Fig. S9. Prior odor or stimulus exposure alone does not influence odor choice.

Line graphs depict mean ± s.e.m of group preference index. Bar graphs depict mean individual preference index, with dots representing individual values per fly. **(A)** Graphic depiction of stimulus or odor exposure procedure and group preference index. **(B-E)** Naïve *Canton-S* but not w^{1118} had a lower average individual preference for side containing EA during the odor-off phase than the choice phase (*Canton-S* $p = 0.004$, w^{1118} p = 0.23). **(F-I)** Sucrose-trained w^{1118}

Journal of Experimental Biology • Supplementary information

but not *Canton-S* had a lower average individual preference for the side containing EA during the odor-off phase than the choice phase (*Canton-S* p = 0.65, w^{1118} p = 0.03). **(J-M)** Average individual preference index did not differ between the odor-off and choice phase in either genotype (*Canton-S* p = 0.33, w^{1118} p =0.09). **(N)** Average individual preference index during the choice phase does not differ between exposure groups or genotype (p = 0.09). Samples, statistical tests and detailed results are found in the S.Figure 9 subsection of Table S3. Experimental replicate and fly exclusion details are found in Table S4.

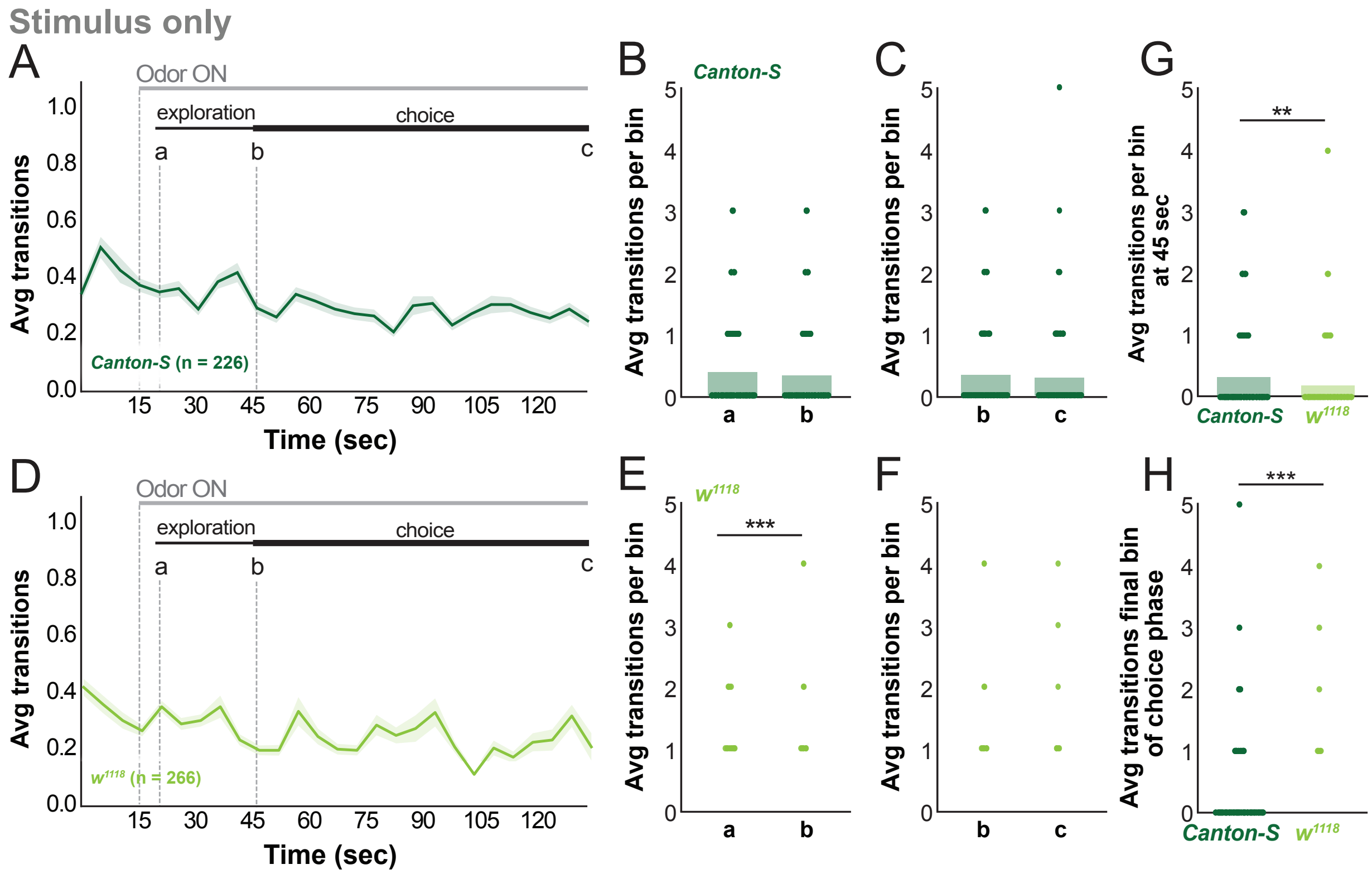

Fig. S10. Transitions made after receiving only odors, sucrose or shock depends on genotype.

(A) Mean ± s.e.m transitions made per 5-second bin across all three *Canton-S* stimulus-only groups (odor-only, sucrose-only, and shock-only) were plotted over time. Bar graphs depict means with dots depicting the values of individual flies. Across *Canton-S* groups, the average number of transitions did not change when comparing the bins that occurred 5 and 30 seconds after odor exposure **(B)** (p = 0.36) or when comparing the bins that occurred 30 and 90 seconds after odor exposure **(C)** (p = 0.31). **(D)** Average transitions per 5-second bin were plotted over time across all w^{1118} stimulus-only groups. **(E)** Across w^{1118} groups, average transitions within the bin that occurred 30 seconds after odor exposure were lower than the bin that occurred 5 seconds after odor exposure (p = 0.0009). **(F)** There was no difference between the average transitions that occurred in the bins 30 seconds and 90 seconds after odor exposure (p = 0.09). **(G)** *Canton-S* flies made more average transitions than w^{1118} in the final bin of the exploration phase (p = 0.007) and in the last bin of the choice phase **(H)** (p = 0.0006). Samples, statistical tests and detailed results are found in the S.Figure 10 subsection of Table S3. Experimental replicate and fly exclusion details are found in Table S4.

Journal of Experimental Biology • Supplementary information

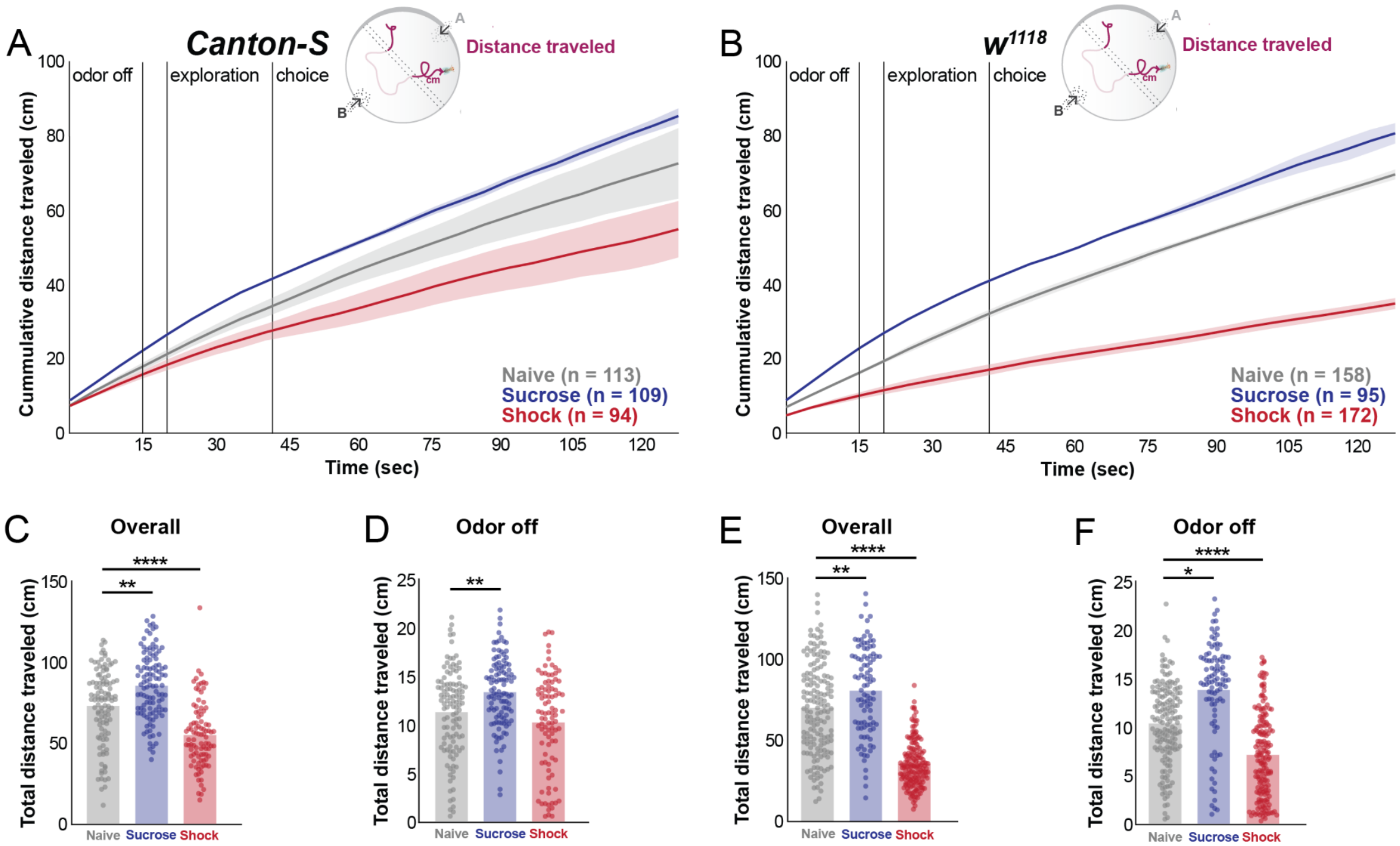

Fig. S11. Locomotor activity depends on conditioning

Distance traveled per region was normalized to the number of flies occupying each region across each video frame. Line graphs depict mean ± s.e.m of total distance travelled and bar graphs depict mean distance travelled per experimental phase, with dots representing individual values per fly. **(A)** Shock-trained *Canton-S* and **(B)** w^{1118} flies travelled less over time than their naïve counterparts, while sucrose-trained flies travelled more than their naïve counterparts. Compared to naïve *Canton-S* flies, shock-trained *Canton-S* travelled less overall **(D)** (p < 0.0001) but not during the odor-off phase **(E)** (p = 0.2), and sucrose-trained *Canton-S* travelled more overall (p = 0.002) and during the odor-off phase (p = 0.001). **(F)** Compared to naïve w^{1118} flies, shock-trained w^{1118} traveled less (p < 0.0001) and sucrose-trained w^{1118} travelled more overall (p = 0.002). **(G)** During the odor-off phase, shock-trained w^{1118} travelled less than naïve w^{1118} flies (p < 0.0001) and sucrose trained w^{1118} traveled more than naïve w^{1118} flies (p = 0.04). Samples, statistical tests and detailed results are found in the S.Figure 11 subsection of Table S3. Experimental replicate and fly exclusion details are found in Table S4.

Journal of Experimental Biology • Supplementary information

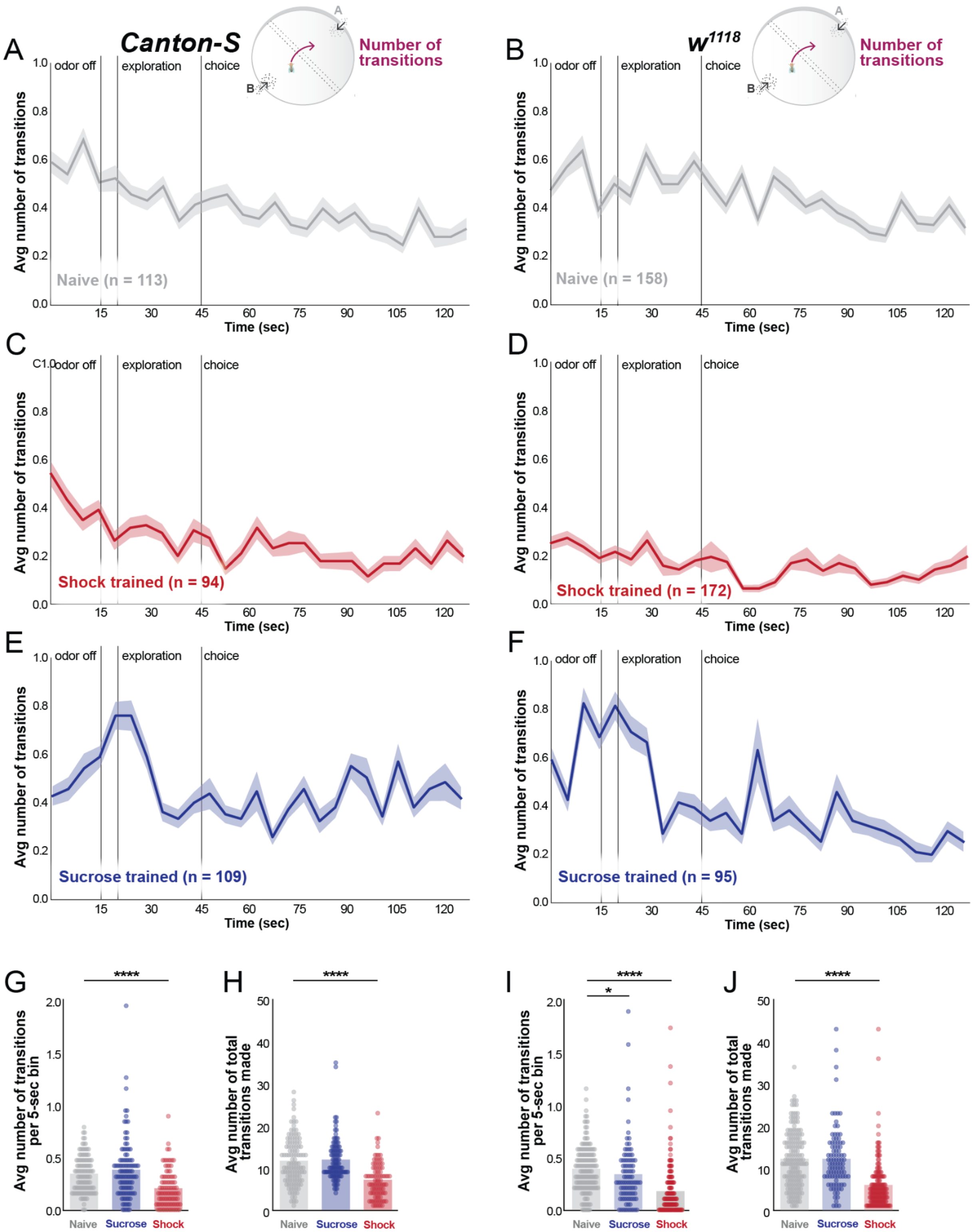

Fig. S12. Shock training decreases average transitions between arena sides.

Line graphs depict mean ± s.e.m of transitions made per 5-second bin over time. Bar graphs depict mean transitions made across flies, with dots representing individual values per fly. **(A-F)** Average transitions over time for each genotype and treatment group (naïve, sucrose-trained, and shock-trained). In *Canton-S* flies, shock reduced the average number of transitions made

Journal of Experimental Biology • Supplementary information

per time bin during the choice phase **(H)** ($p < 0.0001$) and the total transitions made during the experiment **(I)** ($p < 0.0001$). **(J)** During the choice phase, shock-trained and sucrose-trained *w*[1118] flies made fewer average transitions per bin than naïve flies (shock $p < 0.0001$, sucrose $p = 0.03$)**. (K)** Shock-trained *w*[1118] flies made fewer overall transitions than naïve flies ($p < 0.0001$). Samples, statistical tests and detailed results are found in the S.Figure 12 subsection of Table S3. Experimental replicate and fly exclusion details are found in Table S4.

Journal of Experimental Biology • Supplementary information

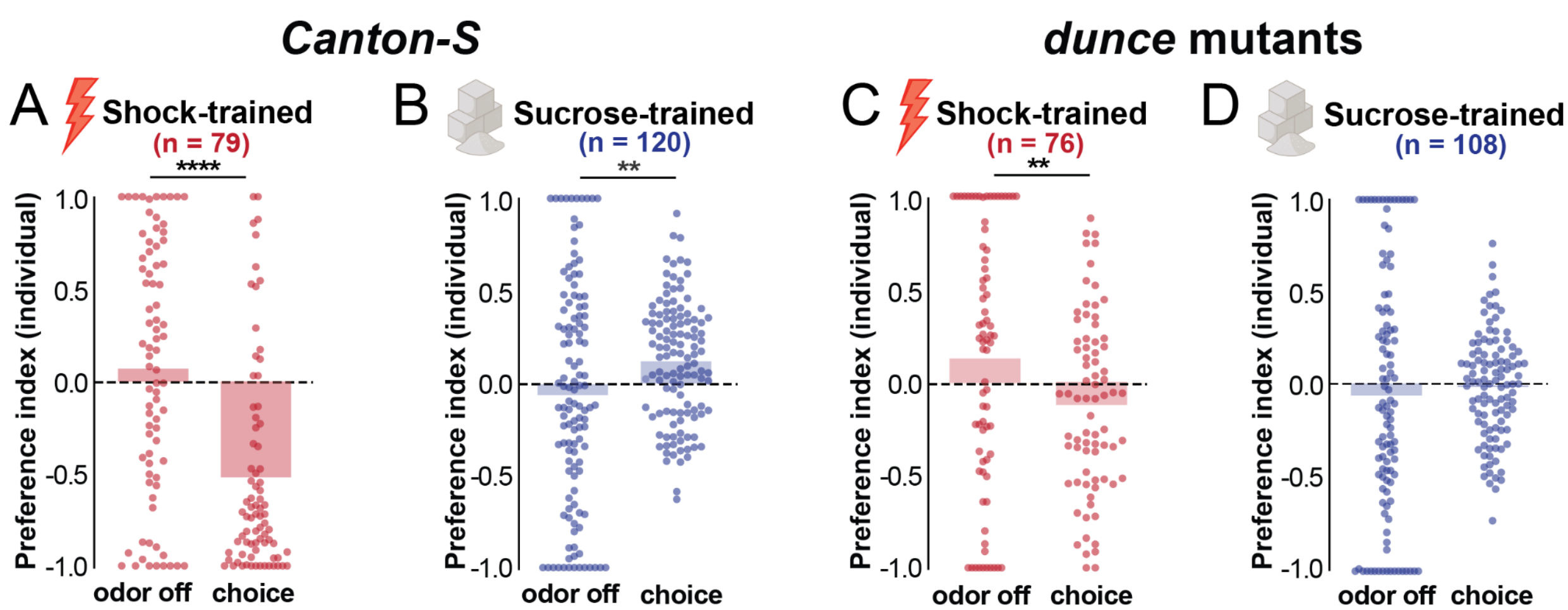

Fig. S13. Within-subject memory in Canton-S and *dunce* strains.

Bar graphs depict mean individual preference index, with dots representing individual values per fly. *Canton-S* flies exhibited both aversive **(A)** and appetitive **(B)** olfactory memory (shock $p < 0.0001$, sucrose $p = 0.009$). **(C)** Shock-trained *dunce* mutants exhibited shock memory ($p = 0.004$), but aversive memory was significantly stronger in *Canton-S* than *dunce* flies (Fig. 5L). **(E)** Sucrose-trained *dunce* mutants did not exhibit appetitive memory ($p = 0.2$). Samples, statistical tests and detailed results are found in the S.Figure 13 subsection of Table S3. Experimental replicate and fly exclusion details are found in Table S4.

Table S1. Two-Choice assay components

COMPONENT	PART ID	VENDOR
PTFE Teflon sheet	3.18 mm thick sheet	CS Hyde
White Acrylic sheet	TL 7328	Various
Clear Plexiglas sheet	3.18 mm thick sheet	Various
Nylon mesh	9318T28	McMaster-Carr
Plastic bonding adhesive	681925	Loctite
Nylon socket head screws	95868A261	McMaster-Carr
Teflon tubes	F015090	Cole Parmer
Teflon tape	23-FEP-.5-5	CS Hyde
Luer to hose barb adapters	MFLX30800-24	Masterflex
Air flowmeter	112-02	Cole-Parmer
Vacuum flowmeter	092-04	Cole-Parmer
3-way valve	411L3212HV	Asko
Swivel-elbow tubes	7880T189	McMaster-Carr
Starvation vials	AS507	Fisher
Luer lock tube coupling	51525K222	McMaster-Carr
Wye connector	5111K542	McMaster-Carr
Threaded lid glass vials	01-672-735	Fisher
Ethyl acetate	S25305	Fisher
Isoamyl acetate	AC150662500	Fisher
Mineral oil	BP26291	Fisher
flyGrRAM Components	N/A	Parts table provided in (Scaplen *et al., 2019*)

Journal of Experimental Biology • Supplementary information

Table S2. Main Figure Statistics and N counts.

Figure	Subfigure	Comparison description	Normality test statistic	Normality test P-value	Levene's test stat	Levene's test P-value	Statistical test	Posthoc analysis	N	P-value	Statistic	Significance	Posthoc P-value	Posthoc Statistic	Posthoc Significance
1	C	Canton-S naïve average transitions in the first and last individual 5-second time bins of exploration	beginning of choice W = 0.5854, end of choice W = 0.5569	odor off p < 0.0001, choice p < 0.0001	N/A	N/A	Wilcoxon matched-pairs signed rank test	N/A	Canton-S naïve (1:512 EA 1:512 IAAc) N = 118, (1:1024 EA 1:1024 IAAc) N = 69, (1:512 EA 1:1024 IAAc) = 113, sucrose-trained = 109, shock-trained = 94. W1118 naïve = 158 , sucrose-trained = 95, shock-trained = 172	0.0007	W = -3240	***	N/A	N/A	N/A
	D	Canton-S naïve average transitions in the first and last individual 5-second time bins of choice phase	beginning of choice W = 0.4741, end of choice W = 0.4370	odor off p < 0.0001, choice p < 0.0001						0.0568	W = -1131	ns			
	L	Naïve Canton-S average preference index during odor-off vs. after choice	odor-off, W = 0.9460, choice W = 0.9675	odor-off, p = 0.0002, choice p = 0.0075						0.1919	W = -901	ns			
	M	Shock-trained Canton-S average preference index during odor-off vs. after choice	odor-off, W = 0.9096, choice W = 0.8895	odor-off, p < 0.0001, choice p < 0.0001						< 0.0001	W = -2481	****			
	N	Sucrose-trained Canton-S average preference index during odor-off vs. after choice	odor-off, W = 0.9031, choice W = 0.9294	odor-off, p < 0.0001, choice p < 0.0001						< 0.0001	W = 2915	****			
	O	Naïve W1118 average preference index during odor-off vs. after choice	odor-off, W = 0.8988, choice W = 0.9605	odor-off, p < 0.0001, choice p = 0.0002						0.1069	W = 1824	ns			
	P	Shock-trained W1118 average preference index during odor-off vs. after choice	odor-off, W = 0.8493, choice W = 0.8544	odor-off, p < 0.0001, choice p < 0.0001						< 0.0001	W = -5294	****			
	Q	Sucrose-trained W1118 average preference index during odor-off vs. after choice	odor-off, W = 0.9212, choice W = 0.9273	odor-off, p < 0.0001, choice p < 0.0001						0.0068	W = 1313	**			
2	B	Canton-S naïve average distance travelled between each arena side during odor off choice and post-choice	K2 = 6.247	p = 0.0440	F = 20.3804	p < 0.0001	Ordinary 2-way ANOVA	N/A	Canton-S naïve (1:512 EA 1:512 IAAc) N = 118, (1:1024 EA 1:1024 IAAc) N = 69, (1:512 EA 1:1024 IAAc) = 113, sucrose-trained = 109, shock-trained = 94. W1118 naïve = 158 , sucrose-trained = 95, shock-trained = 172	Arena side factor p = 0.7896, phase factor p < 0.0001, interaction p = 0.2402	Arena side factor F(1, 672) = 0.0713, phase factor F(2, 672) = 30.96, interaction F(2, 672) = 1.429	Arena side factor (ns) , phase factor (****), interaction (ns)	N/A	N/A	N/A
	D	W1118 naïve average distance travelled between each arena side during odor off choice and post-choice	K2 = 40.91	p < 0.0001	F = 22.5126	p = < 0.0001	Ordinary 2-way ANOVA	N/A		Arena side factor p = 0.2938, phase factor p < 0.0001, interaction p = 0.8231	Arena side factor F(1, 942) = 1.104, phase factor F(2, 942) = 24.53, interaction F(2, 942) = 0.1947	Arena side factor (ns) , phase factor (****), interaction (ns)	N/A	N/A	N/A
	F	Canton-S shock-trained average distance travelled between each arena side during odor off, choice and post-choice	K2 = 41.46	p < 0.0001	F = 29.3084	p < 0.0001	Ordinary 2-way ANOVA	Tukey's multiple comparisons test		Arena side factor p < 0.0001, phase factor p < 0.0001, interaction p = 0.3114	Arena side factor F(1, 558) = 33.51, phase factor F(2, 558) = 37.41, interaction F(2, 558) = 1.169	Arena side factor (****) , phase factor (****), interaction (ns)	odor off p = 0.0346, exploration p = 0.0002, choice p < 0.0001	odor off q (558) = 2.996, exploration q (558) = 5.290, choice q (558) = 5.894	odor off (*), choice (***), post-choice (****)
	H	W1118 shock-trained average distance travelled between each arena side during odor off, choice and post-choice	K2 = 214.8	p < 0.0001	F = 44.9204	p < 0.0001	Ordinary 2-way ANOVA	Tukey's multiple comparisons test		Arena side factor p < 0.0001 , phase factor p < 0.0001, interaction p = 0.6057	Arena side factor F(1, 1026) = 35.69, phase factor F(2, 1026) = 56.77, interaction F(2, 1026) = 0.5016	Arena side factor (****) , phase factor (****), interaction (ns)	odor off p = 0.0028, exploration p = 0.0022 choice p < 0.0001	odor off q (1026) = 4.253, exploration q (1026) = 4.347, choice q (1026) = 6.033	odor off (**), exploration (**), choice (****)
	J	Canton-S sucrose-trained average distance travelled between each arena side during odor off, choice and post-choice	K2 = 1.032	p = 0.5968	F = 28.5511	p = < 0.0001	Ordinary 2-way ANOVA	Tukey's multiple comparisons test		Arena side factor p = 0.0165, phase factor p < 0.0001, interaction p < 0.0001	Arena side factor F(1, 648) = 5.782, phase factor F(2, 648) = 39.52, interaction F(2, 648) = 11.74	Arena side factor (*), phase factor (****), interaction (****)	odor off p = 0.0151, exploration p = 0.0156, choice p < 0.0001	odor off q (648) = 3.378, exploration q (648) = 3.882, choice q (648) = 5.789	odor off (*), exploration (*), choice (****)
	L	W1118 sucrose-trained average distance travelled between each arena side during odor off, choice and post-choice	K2 = 9.439	p = 0.0089	F = 29.0275	p = < 0.0001	Ordinary 2-way ANOVA	Tukey's multiple comparisons test		Arena side factor p < 0.0001 , phase factor p < 0.0001, interaction p = 0.1455	Arena side factor F(1, 564) = 20.04, phase factor F(2, 564) = 36.45, interaction F(2, 564) = 1.934	Arena side factor (****) , phase factor (****), interaction (ns)	odor off p = 0.2214, exploration p = < 0.0002 choice p = 0.0095	odor off q (564) = 1.731, exploration q (564) = 5.341, choice q (564) = 3.683	odor off (ns), exploration (***), choice (**)
3	B	Canton-S naïve average bout duration between each arena side during odor off choice and post-choice	K2 = 45.31	p < 0.0001	F = 1.5834	p = 0.1625	Ordinary 2-way ANOVA	N/A	Canton-S naïve (1:512 EA 1:512 IAAc) N = 118, (1:1024 EA 1:1024 IAAc) N = 69, (1:512 EA 1:1024 IAAc) = 113, sucrose-trained = 109, shock-trained = 94. W1118 naïve = 158 , sucrose-trained = 95, shock-trained = 172	Arena side factor p = 0.4545, phase factor p = 0.1078, interaction p = 0.0012	Arena side factor F(1, 672) = 0.5600, phase factor F(2, 672) = 2.235, interaction F(2, 672) = 6.764	Arena side factor (ns) , phase factor (ns), interaction (**)	N/A	N/A	N/A
	D	W1118 naïve average bout duration between each arena side during odor off choice and post-choice	K2 = 86.78	p < 0.0001	F = 4.2688	p = 0.0007	Ordinary 2-way ANOVA	N/A		Arena side factor p = 0.9444, phase factor p = 0.5660 interaction p = 0.3423	Arena side factor F(1, 942) = 0.00486, phase factor F(2, 942) = 0.5696, interaction F(2, 942) = 1.073	Arena side factor (ns) , phase factor (ns), interaction (ns)	N/A	N/A	N/A
	F	Canton-S shock-trained average bout duration between each arena side during odor off, choice and post-choice	K2 = 45.48	p < 0.0001	F = 11.255	p < 0.0001	Ordinary 2-way ANOVA	Tukey's multiple comparisons test		Arena side factor p < 0.0001, phase factor p = 0.4608, interaction p < 0.0001	Arena side factor F(1, 588) = 87.25, phase factor F(2, 558) = 0.7758 interaction F(2, 558) = 23.85	Arena side factor (****) , phase factor (ns), interaction (****)	odor off p = 0.2883, exploration p < 0.0001, choice p < 0.0001	odor off q (558) = 1.503, exploration q (558) = 6.264, choice q (558) = 15.11	odor off (ns), exploration (****), choice (****)
	H	W1118 shock-trained average bout duration between each arena side during odor off, choice and post-choice	K2 = 1941	p < 0.0001	F = 9.0052	p < 0.0001	Ordinary 2-way ANOVA	Tukey's multiple comparisons test		Arena side factor p < 0.0001 , phase factor p = 0.5437, interaction p < 0.0001	Arena side factor F(1, 1026) = 59.06, phase factor F(2 , 1026) = 0.6097, interaction F(2, 1026) = 19.96	Arena side factor (****) , phase factor (ns), interaction (****)	odor off p = 0.2600, exploration p = 0.0078 choice p < 0.0001	odor off q (1026) = 1.594, exploration q (1026) = 3.769, choice q (1026) = 13.46	odor off (ns), expl,oration(**), choice (****)
	J	Canton-S sucrose-trained average bout duration between each arena side during odor off, choice and post-choice	K2 = 31.45	p < 0.0001	F = 11.7195	p < 0.0001	Ordinary 2-way ANOVA	Tukey's multiple comparisons test		Arena side factor p = < 0.0001, phase factor p = 0.5179, interaction p < 0.0001	Arena side factor F(1, 648) = 31.20, phase factor F(2, 648) = 0.6587, interaction F(2, 648) = 30.59	Arena side factor (****) , phase factor (ns), interaction (****)	odor off p = 0.0394, exploration p = 0.0058, choice p < 0.0001	odor off q (648) = 2.919, exploration q (648) = 3.916, choice q (648) = 12.69	odor off (*), exploration (**), choice (****)
	L	W1118 sucrose-trained average bout duration between each arena side during odor off, choice and post-choice	K2 = 852.2	p < 0.0001	F = 2.9371	p = 0.01254	Ordinary 2-way ANOVA	Tukey's multiple comparisons test		Arena side factor p < 0.0001 , phase factor p = 0.0536, interaction p = 0.0029	Arena side factor F(1, 564) = 30.65, phase factor F(2, 564) = 2.941, interaction F(2, 564) = 5.912	Arena side factor (****) , phase factor (ns), interaction (**)	odor off p = 0.6688, exploration p < 0.0001 choice p < 0.0001	odor off q (564) = 0.6053, exploration q (564) = 5.904, choice q (564) = 7.051	odor off (ns), exploration (****), choice (****)
4	B	Canton-S naïve average U-turns per cm between arena sides after choice	EA side W = 0.6089, IAAc side W = 0.7644	EA side p < 0.0001, IAAc side p < 0.0001	N/A	N/A	Wilcoxon matched-pairs signed rank test	N/A	Canton-S naïve = 113	0.1592	W = 473	ns	N/A	N/A	N/A
	C	Canton-S shock-trained average U-turns per cm between arena sides after choice	no shock side W = 0.8184, shock side W = 0.4850	no shock side p < 0.0001, shock side p < 0.0001					Canton-S shock-trained = 94	< 0.0001	W = -1538	****			
	D	Canton-S sucrose-trained average U-turns per cm between arena sides after choice	H20 side W = 0.6249, sucrose side W = 0.7589	H20 side p < 0.0001, sucrose side p < 0.0001					Canton-S sucrose-trained = 109	0.028	W = 966	*			
	E	W1118 naïve average U-turns per cm between arena sides after choice	EA side W = 0.5867, IAAc side W = 0.5908	EA side p < 0.0001, IAAc side p < 0.0001					W1118 naïve = 158	0.324	W = -585	ns			
	F	W1118 shock-trained average U-turns per cm between arena sides after choice	no shock side W = 0.6424, shock side W = 0.4094	no shock side p < 0.0001, shock side p < 0.0001					W1118 shock-trained =172	0.1583	W = -397	ns			
	G	W1118 sucrose-trained average U-turns per cm between arena sides after choice	H20 side W = 0.4458, sucrose side W = 0.7450	H20 side p < 0.0001, sucrose side p < 0.0001					W1118 sucrose-trained = 95	0.0032	W = 753	**			
	I	Canton-S naïve average distance to odor port on each arena side after choice	EA side W = 0.9902, IAAc side W = 0.9821	EA side p = 0.6380, IAAc side W = 0.1730			paired T-test (two-tailed)		Canton-S naïve = 98	0.1272	t(97) = 1.538	ns			
	J	Canton-S shock-trained average distance to odor port on each arena side after choice	no shock side W = 0.9771, shock side W = 0.9766	no shock side p = 0.0974, shock side p = 0.0896			paired T-test (one-tailed)		Canton-S shock-trained = 94	< 0.0001	t(93) = 10.62	****			
	K	Canton-S sucrose-trained average distance to odor port on each arena side after choice	H20 side W = 0.9715, sucrose side W = 0.7964	H20 side p = 0.0240, sucrose side p < 0.0001			Wilcoxon matched-pairs signed rank test		Canton-S sucrose-trained = 104	< 0.0001	W = -3784	****			
	L	W1118 naïve average distance to odor port on each arena side after choice	EA side W = 0.9793, IAAc side W = 0.9757	EA side p = 0.0249, IAAc side W = 0.0099			Wilcoxon matched-pairs signed rank test		W1118 naïve = 138	0.573	W = 533	ns			
	M	W1118 shock-trained average distance to odor port on each arena side after choice	no shock side W = 0.9938, shock side W = 0.9845	no shock side p = 0.7179, shock side p = 0.1403			paired T-test (one-tailed)		W1118 shock-trained =124	< 0.0001	t(123) = 4.298	****			
	N	W1118 sucrose-trained average distance to odor port on each arena side after choice	H20 side W = 0.9930, sucrose side W = 0.9942	H20 side p = 0.9239, sucrose side p = 0.9576			paired T-test (one-tailed)		W1118 sucrose-trained = 88	0.0358	t(87) = 1.824	*			
	P	Canton-S naïve total stops close to port on each arena side after choice	EA side W = 6418, IAAc side W = 0.7438	EA side p = < 0.0001, IAAc side p < 0.0001			Wilcoxon matched-pairs signed rank test		Canton-S naïve = 113	0.052	W = 686	ns			
	Q	Canton-S shock-trained total stops close to port on each arena side after choice	no shock side W = 0.7687, shock side W = 0.3338	no shock side p < 0.0001, shock side p < 0.0001					Canton-S shock-trained = 94	< 0.0001	W = -1342	****			
	R	Canton-S sucrose-trained total stops close to port on each arena side after choice	H20 side W = 0.4874, sucrose side W = 0.6859	H20 side p < 0.0001, sucrose side p < 0.0001					Canton-S sucrose-trained = 109	< 0.0001	W = 853	****			
	S	W1118 naïve total stops close to port on each arena side after choice	EA side W = 6102, IAAc side W = 0.5152	EA side p = < 0.0001, IAAc side p < 0.0001					W1118 naïve = 158	0.9043	W = -53	ns			
	T	W1118 shock-trained total stops close to port on each arena side after choice	no shock side W = 0.5673, shock side W = 0.2926	no shock side p < 0.0001, shock side p < 0.0001					W1118 shock-trained =172	0.0002	W = -976	***			
	U	W1118 sucrose-trained total stops close to port on each arena side after choice	H20 side W = 0.6104, sucrose side W = 0.7223	H20 side p < 0.0001, sucrose side p < 0.0001					W1118 sucrose-trained = 95	0.0002	W = 821	***			
5	C	Shock-trained Canton-S vs. Dunce average post-choice preference index	Canton-S W = 0.7955, *dunce* W = 0.9801	Canton-S p < 0.0001, *dunce* p < 0.2767	F = 0.0001	p = 0.99	Mann-Whiteney U-test	N/A	Canton-S sucrose-trained = 120, Dunce sucrose-trained = 108, Canton-S shock trained = 79, Dunce shock trained = 76	< 0.0001	U = 1554	****	N/A	N/A	N/A
	D	Shock-trained Canton-S vs. Dunce average post-choice U-turns per cm. travelled	Canton-S W = 0.8917, *dunce* W = 0.8446	Canton-S p < 0.0001, *dunce* p < 0.0001	F = 4.7707	p = 0.03				0.019	U = 2439	*			
	E	Shock-trained Canton-S vs. Dunce average distance to sucrose paired odor side after choice	Canton-S W = 0.9497, *dunce* W = 0.9722	Canton-S p = 0.0041, *dunce* p = 0.0938	F = 2.5894	p = 0.1097				< 0.0001	U = 1787	****			
	F	Shock-trained Canton-S vs. Dunce total stops close to sucrose-paired port after choice	Canton-S W = 0.8305, *dunce* W = 0.7936	Canton-S p < 0.0001, *dunce* p < 0.0001	F = 18.5324	p < 0.0001				0.0009	U = -1032	***			
	G	Sucrose-trained Canton-S vs. Dunce average post-choice preference index	Canton-S W = 0.9852, *dunce* W = 0.9938	Canton-S p = 0.2126, *dunce* p = 0.8987	F = 3.2411	p = 0.07315	Welch's t-test			0.0014	U = 4998	**			
	H	Sucrose-trained Canton-S vs. Dunce average post-choice U-turns per cm. travelled	Canton-S W = 0.9216, *dunce* W = 0.8640	Canton-S p < 0.0001, *dunce* p < 0.0001	F = 0.02051	p = 0.8863	Mann-Whiteney U-test			0.0026	U =5100	**			
	I	Sucrose-trained Canton-S vs. Dunce average distance to sucrose paired odor side after choice	Canton-S W = 0.9828, *dunce* W = 0.9906	Canton-S p = 0.1291, *dunce* p = 0.6652	F = 5.9311	p = 0.01565	Welch's t-test			< 0.0001	U = 4397	****			
	J	Sucrose-trained Canton-S vs. Dunce total stops close to sucrose-paired port after choice	Canton-S W = 0.8263, *dunce* W = 0.7350	Canton-S p < 0.0001, *dunce* p < 0.0001	F = 31.199	p < 0.0001	Mann-Whiteney U-test			< 0.0001	U = 3591	****			

Available for download at
https://journals.biologists.com/jeb/article-lookup/doi/10.1242/jeb.253164#supplementary-data

Table S3. Supplemental figure statistics and N counts.

Figure	Subfigure	Comparison description	Normality test statistic	Normality test P-value	Levene's test stat	Levene's test P-value	Statistical test	Posthoc analysis	N	P-value	Statistic	Significance	Posthoc P-value	Posthoc Statistic	Posthoc Significance
S.4	C	Canton-S naïve average transitions in the first and last individual 1-second time bins of exploration	beginning of choice W = 0.3678, end of choice W = 0.2350	odor off p < 0.0001, choice p < 0.0001	N/A	N/A	Wilcoxen matched-pairs signed rank test	N/A	(1:512 EA 1:512 IAAc) N = 118, (1:1024 EA 1:1024 IAAc), N = 69, (1:512 EA 1:1024 IAAc) = 113	0.0233	W = -424	*	N/A	N/A	N/A
	D	Canton-S naïve average transitions in the first and last individual 1-second time bins of choice phase	beginning of choice W = 0.2427, end of choice W = 0.1797	odor off p < 0.0001, choice p < 0.0001						0.4119	W = -69	ns			
S.5	C	(1:512 EA 1:512 IAAc) Canton-S average preference index between odor off and choice phases	odor off W = 0.8809, choice W = 0.9116	odor off p < 0.0001, choice p < 0.0001	N/A	N/A	Wilcoxen matched-pairs signed rank test	N/A	(1:512 EA 1:512 IAAc) N = 118, (1:1024 EA 1:1024 IAAc) N = 69, (1:512 EA 1:1024 IAAc) = 113, W1118 (1:512 EA 1:1024 IAAc) N = 158	<0.0001	W = 3361	****	N/A	N/A	N/A
	E	(1:1024 EA 1:1024 IAAc) Canton-S average preference index between odor off and choice phases	odor off W = 0.7985, choice W = 0.8355	odor off p < 0.0001, choice p < 0.0001						0.0158	W = 735	*			
	G	(1:512 EA 1:1024 IAAc) Canton-S average preference index between odor off and choice phases	odor off W = 0.9724, choice W = 0.9573	odor off p < 0.0001, choice p = 0.0004						0.3343	W = 668	ns			
	I	(1:512 EA 1:1024 IAAc) W1118 average preference index between odor off and choice phases	odor off W = 0.9032, choice W = 0.9696	odor off p < 0.0001, choice p < 0.0001						0.8015	W = 286	ns			
S.7	A	Canton-S EA shock paired preference index odor-off vs. choice phase arena 1	odor off W = 0.6525, choice W = 0.9204	odor off p = 0.0002, choice p = 0.3606	N/A	N/A	Wilcoxon matched-pairs signed rank test	N/A	Canton-S shock-trained = 94 Canton-S sucrose trained = 109	0.4258	W = 15	ns	N/A	N/A	N/A
	B	Canton-S EA shock paired preference index odor-off vs. choice phase arena 2	odor off W = 0.9622, choice W = 0.8928	odor off p = 0.8104, choice p = 0.8928			paired t-test			0.1265	t(9) = 1.684	ns			
	C	Canton-S EA shock paired preference index odor-off vs. choice phase arena 3	odor off W = 0.9181, choice W = 0.9083	odor off p = 0.3412, choice p = 0.2693			paired t-test			0.0469	t(9) = 2.301	*			
	D	Canton-S EA shock paired preference index odor-off vs. choice phase arena 4	odor off W = 0.8845, choice W = 0.9229	odor off p = 0.1470, choice p = 0.3820			paired t-test			0.1015	t(9) = 1.824	ns			
	E	Canton-S EA shock paired preference index odor-off vs. choice phase arena 5	odor off W = 0.8653, choice W = 0.9100	odor off p = 0.1688, choice p = 0.3960			paired t-test			0.4484	t(6) = 0.8111	ns			
	F	Canton-S IAAc shock paired preference index odor-off vs. choice phase arena 1	odor off W = 0.8476, choice W = 0.9284	odor off p = 0.0701, choice p = 0.4659			paired t-test			0.0163	t(8) = 3.032	*			
	G	Canton-S IAAc shock paired preference index odor-off vs. choice phase arena 2	odor off W = 0.9517, choice W = 0.5788	odor off p = 0.6881, choice p < 0.0001			Wilcoxon matched-pairs signed rank test			0.0039	W = -45	**			
	H	Canton-S IAAc shock paired preference index odor-off vs. choice phase arena 3	odor off W = 0.8298, choice W = 0.9669	odor off p = 0.0591, choice p = 0.8715			paired t-test			0.3212	t(7) = 1.067	ns			
	I	Canton-S IAAc shock paired preference index odor-off vs. choice phase arena 4	odor off W = 0.8377, choice W = 0.9315	odor off p = 0.0414, choice p = 0.4628			Wilcoxon matched-pairs signed rank test			0.084	W = -35	ns			
	J	Canton-S IAAc shock paired preference index odor-off vs. choice phase arena 5	odor off W = 0.7648, choice W = 0.9255	odor off p = 0.0080, choice p = 0.4396			Wilcoxon matched-pairs signed rank test			0.0039	W = -45	**			
	K	Canton-S EA sucrose paired preference index odor-off vs. choice phase arena 1	odor off W = 0.9105, choice W = 0.9697	odor off p = 0.2846, choice p = 0.8876			paired t-test			0.7418	t(9) = 0.3398	ns			
	L	Canton-S EA sucrose paired preference index odor-off vs. choice phase arena 2	odor off W = 0.9040, choice W = 0.6764	odor off p = 0.3560, choice p = 0.0020			Wilcoxon matched-pairs signed rank test			0.2188	W = 16	ns			
	M	Canton-S EA sucrose paired preference index odor-off vs. choice phase arena 3	odor off W = 0.9378, choice W = 0.9038	odor off p = 0.5287, choice p = 0.2410			paired t-test			< 0.0001	t(9) = 9.606	****			
	N	Canton-S EA sucrose paired preference index odor-off vs. choice phase arena 4	odor off W = 0.8437, choice W = 0.9814	odor off p = 0.0488, choice p = 0.9723			Wilcoxon matched-pairs signed rank test			0.0098	W = 49	**			
	O	Canton-S EA sucrose paired preference index odor-off vs. choice phase arena 5	odor off W = 0.7957, choice W = 0.8959	odor off p = 0.0129, choice p = 0.1975			Wilcoxon matched-pairs signed rank test			0.0098	W = 49	**			
	P	Canton-S EA sucrose paired preference index odor-off vs. choice phase arena 6	odor off W = 0.8241, choice W = 0.9344	odor off p = 0.0284, choice p = 0.4929			Wilcoxon matched-pairs signed rank test			0.0137	W = 47	*			
	Q	Canton-S IAAc sucrose paired preference index odor-off vs. choice phase arena 1	odor off W = 0.8204, choice W = 0.9260	odor off p = 0.0256, choice p = 0.4098			Wilcoxon matched-pairs signed rank test			0.0059	W = 51	**			
	R	Canton-S IAAc sucrose paired preference index odor-off vs. choice phase arena 2	odor off W = 0.8920, choice W = 0.9599	odor off p = 0.1787, choice p = 0.7725			paired t-test			0.1685	t(9) = 1.498	ns			
	S	Canton-S IAAc sucrose paired preference index odor-off vs. choice phase arena 3	odor off W = 0.8685, choice W = 0.8783	odor off p = 0.0959, choice p = 0.1247			paired t-test			0.2979	t(9) = 1.105	ns			
	T	Canton-S IAAc sucrose paired preference index odor-off vs. choice phase arena 4	odor off W = 0.8995, choice W = 0.8958	odor off p = 0.3279, choice p = 0.3062			paired t-test			0.0637	t(6) = 2.270	ns			
	U	Canton-S IAAc sucrose paired preference index odor-off vs. choice phase arena 5	odor off W = 0.7970, choice W = 0.8744	odor off p = 0.0133 choice p = 0.1124			Wilcoxon matched-pairs signed rank test			> 0.9999	W = -1	ns			
	V	Canton-S IAAc sucrose paired preference index odor-off vs. choice phase arena 6	odor off W = 0.9648, choice W = 0.8818	odor off p = 0.8412 choice p = 0.3175			paired t-test			0.8851	t(4) = 0.1540	ns			
S.8	B	EA-shock paired Canton-S preference index	odor off W = 0.9130, choice W = 0.8480	odor off p = 0.0017, choice p <0.0001	N/A	N/A	Wilcoxon matched-pairs signed rank test	N/A	CS shock-trained EA sucrose paired = 48	< 0.0001	W = -814	****	N/A	N/A	N/A
	C	IAAc-shock paired Canton-S preference index	odor off W = 0.8985, choice W = 0.9122	odor off p = 0.0007, choice p <0.0021					CS shock-trained IAAc sucrose paired = 46	0.0212	W = -371	*			
	E	EA-shock paired W1118 preference index	odor off W = 0.8285, choice W = 0.8816	odor off p < 0.0001, choice p <0.0001					W1118 shock-trained EA sucrose paired = 80	0.0156	W = -846	*			
	F	IAAc-shock paired W1118 preference index	odor off W = 0.8535, choice W = 0.8207	odor off p < 0.0001, choice p <0.0001					W1118 shock-trained IAAc sucrose paired = 92	0.0004	W = -1645	***			
	H	EA-suc paired Canton-S preference index	odor off W = 0.8968, choice W = 0.8458	odor off p = 0.0001, choice p <0.0001					CS sucrose-trained EA sucrose paired = 57	< 0.0001	W = 1235	****			
	I	IAAc-suc paired Canton-S preference index	odor off W = 0.8982, choice W = 0.9700	odor off p = 0.0003, choice p = 0.2110					CS sucrose-trained IAAc sucrose paired = 52	0.2213	W = 166	ns			
	K	EA-suc paired W1118 preference index	odor off W = 0.9226, choice W = 0.9036	odor off p = 0.0029, choice p = 0.0006					W1118 sucrose-trained EA sucrose paired = 50	0.179	W = 187	ns			
	L	IAAc-suc paired W1118 preference index	odor off W = 0.9134, choice W = 0.9375	odor off p = 0.0025, choice p = 0.0174					W1118 sucrose-trained IAAc sucrose paired = 45	0.0041	W = 463	**			
S.9	C	CantonS odor only preference for EA	odor off W = 0.8541, choice W = 0.9658	odor off p < 0.0001, choice p = 0.0309	N/A	N/A	Wilcoxon matched-pairs signed rank test	N/A	Canton-S odor only N = 80, W1118 odor only N = 66, Canton-S sucrose only N = 80, W1118 sucrose only N = 75, Canton-S shock only N = 97, W1118 shock only N = 94	0.0038	W = 1194	**	N/A	N/A	N/A
	E	W1118 odor only preference for EA	odor off W = 0.8558, choice W = 0.8639	odor off p < 0.0001, choice p < 0.0001						0.2317	W = 360	ns			
	G	CantonS sucrose-only preference for EA	odor off W = 0.8823, choice W = 0.9826	odor off p < 0.0001, choice p = 0.3503						0.6486	W = -192	ns			
	I	W1118 sucrose-only preference for EA	odor off W = 0.8580, choice W = 0.9194	odor off p < 0.0001, choice p = 0.0001						0.0305	W = 817	*			
	K	CantonS shock-only preference for EA	odor off W = 0.9050, choice W = 0.9857	odor off p < 0.0001, choice p = 0.3779						0.0552	W = 1065	ns			
	M	W1118 shock-only preference for EA	odor off W = 0.8085, choice W = 0.9154	odor off p < 0.0001, choice p < 0.0001						0.3373	W = -487	ns			
	N	Comparing choice phase preference for EA across conditions	CS odor only K2 = 13.91, W1118 odor only K2 = 120.1, CS suc only K2 = 0.4585, W1118 suc only K2 = 35.43, CS shock only K2 = 1.549, W1118 shock only K2 = 49.15	CS odor only p = 0.0010, W1118 odor only p < 0.0001, CS suc only K2 = 0.7951, W1118 suc only p < 0.0001, CS shock only p = 0.4609, W1118 shock only p = 0.9154	F = 15.475	p < 0.0001	Kruskal-Wallis test			0.0887	H = 9.561	ns			
S.10	B	CantonS stimulus only (all stimulus only groups) average transitions per 5 second bin between the first and last bins of exploration	1st bin W = 0.6040, last bin W = 0.5477	1st bin p < 0.0001, last bin p < 0.0001	N/A	N/A	Wilcoxon matched-pairs signed rank test	N/A	Canton-S odor only N = 80, W1118 odor only N = 66, Canton-S sucrose only N = 80, W1118 sucrose only N = 75, Canton-S shock only N = 97, W1118 shock only N = 94	0.3581	W = -425	ns	N/A	N/A	N/A
	C	CantonS stimulus only (all stimulus only groups) average transitions per 5 second bin between the first and last bins of choice	1st bin W = 0.5477, last bin W = 0.4963	1st bin p < 0.0001, last bin p < 0.0001						0.3051	W = -460	ns			
	E	W1118 stimulus only (all stimulus only groups) average transitions per 5 second bin between the first and last bins of exploration	1st bin W = 0.6029, last bin W = 0.4110	1st bin p < 0.0001, last bin p < 0.0001						0.0009	W = -1131	***			
	F	W1118 stimulus only (all stimulus only groups) average transitions per 5 second bin between the first and last bins of choice	1st bin W = 0.4110, last bin W = 0.1103	1st bin p < 0.0001, last bin p < 0.0001						0.0858	W = -344	ns			
	G	Canton-S vs W1118 average transitions made during the last bin of the exploration phase	CS W = 0.5444, W1118 W = 0.3884	CS p < 0.0001, W1118 p < 0.0001			Mann-Whitney test			0.0072	U = 30558	**			
	H	Canton-S vs W1118 average transitions made during the last bin of the choice phase	CS W = 0.4963, W1118 W = 0.1103	CS p < 0.0001, W1118 p < 0.0001						0.0006	U = 26772	***			
S.11	C	Canton-S total total distance travelled between condition	Naïve K2 = 6.292, sucrose K2 = 3.619, shock K2 = 12.91	naïve p = 0.0430, sucrose p = 0.1637, shock p = 0.0016	F = 1.2033	p = 0.3016	Kruskal-Wallis test	Dunn's test	Canton-S naïve = 113, sucrose-trained = 109, shock-trained = 94. W1118 naïve = 158 , sucrose-trained = 95, shock-trained = 172	< 0.0001	H = 79.97	****	naïve vs. sucrose p = 0.0019, naïve vs. shock p < 0.0001, sucrose vs. shock p < 0.0001	naïve vs. sucrose z = 3.423, naïve vs. shock z = 5.666, sucrose vs. shock z = 8.885	naïve vs. sucrose **, naïve vs. shock ****, sucrose vs. shock ****
	D	Canton-S distance travelled during the odor-off phase between conditions	Naïve K2 = 2.631, sucrose K2 = 2.048, shock K2 = 5.944	naïve p = 0.2684, sucrose p = 0.3591, shock p = 0.0512	F = 4.007	p = 0.0191	1-way ANOVA	Tukey's test		< 0.0001	F = 13.65	****	naïve vs. sucrose p = 0.0013, naïve vs. shock p =0.1998, sucrose vs. shock p < 0.0001	naïve vs. sucrose q = 5.004, naïve vs. shock q = 2.431, sucrose vs. shock z = 7.813	naïve vs. sucrose **, naïve vs. shock ns, sucrose vs. shock ****
	E	W1118 total total distance travelled between condition	Naïve K2 = 8.157, sucrose K2 = 4.592, shock K2 = 14.83	naïve p = 0.0169, sucrose p = 0.1007, shock p = 0.0006	F = 46.8549	p < 0.0001	Kruskal-Wallis test	Dunn's test		< 0.0001	H = 184.5	****	naïve vs. sucrose p = 0.0019, naïve vs. shock p < 0.0001, sucrose vs. shock p < 0.0001	naïve vs. sucrose z = 3.423, naïve vs. shock z = 5.666, sucrose vs. shock z = 8.885	naïve vs. sucrose **, naïve vs. shock ****, sucrose vs. shock ****
	F	W1118 distance travelled during the odor-off phase between conditions	Naïve K2 = 1.190, sucrose K2 = 11.51, shock K2 = 14.35	naïve p = 0.5514, sucrose p = 0.0032, shock p = 0.0008	F = 0.8451	p = 0.4301				< 0.0001	H = 108.2	****	naïve vs. sucrose p = 0.0416, naïve vs. shock p < 0.0001, sucrose vs. shock p < 0.0001	naïve vs. sucrose z = 2.461, naïve vs. shock z = 10.89, sucrose vs. shock z = 11.89	naïve vs. sucrose *, naïve vs. shock ****, sucrose vs. shock ****
S.12	G	Canton-S average trasitions per bin in the choice phase	Naïve K2 = 3.880, sucrose K2 = 57.04, shock K2 = 11.94	naïve p = 0.1437, sucrose p < 0.0010, shock p = 0.0026	F = 4.6163	p = 001057	Kruskal-Wallis test	Dunn's test	Canton-S naïve = 113, sucrose-trained = 109, shock-trained = 94. W1118 naïve = 158 , sucrose-trained = 95, shock-trained = 172	< 0.0001	H = 38.31	****	naïve vs. sucrose p = >0.9999, naïve vs. shock p < 0.0001, sucrose vs. shock p < 0.0001	naïve vs. sucrose z = 0.05715, naïve vs. shock z = 5.429, sucrose vs. shock z = 5.439	naïve vs. sucrose ns, naïve vs. shock ****, sucrose vs. shock ****
	H	Canton-S total transitions	Naïve K2 = 5.020, sucrose K2 = 41.54, shock K2 = 137.7	naïve p = 0.0813, sucrose p < 0.0001, shock p < 0.0001	F = 4.5251	p = 001155				< 0.0001	H = 53.68	****	naïve vs. sucrose p = >0.9999, naïve vs. shock p < 0.0001, sucrose vs. shock p < 0.0001	naïve vs. sucrose z = 0.6004, naïve vs. shock z = 6.153, sucrose vs. shock z = 6.675	naïve vs. sucrose ns, naïve vs. shock **, sucrose vs. shock ****
	I	W1118 average trasitions per bin in the choice phase	Naïve K2 = 4.558, sucrose K2 = 66.36, shock K2 = 20.28	naïve p = 0.1024, sucrose p < 0.0001, shock p < 0.0001	F = 2.4853	p = 0.08452				< 0.0001	H = 102.9	****	naïve vs. sucrose p = 0.0325, naïve vs. shock p < 0.0001, sucrose vs. shock p < 0.0001	naïve vs. sucrose z = 2.548, naïve vs. shock z = 9.461, sucrose vs. shock z = 5.568	naïve vs. sucrose *, naïve vs. shock ****, sucrose vs. shock ****
	J	W1118 total transitions	Naïve K2 = 9.999, sucrose K2 = 66.80, shock K2 = 138.8	naïve p = 0.0067, sucrose p < 0.0001, shock p < 0.0001	F = 7.142	p = 0.0009				< 0.0001	H = 92.84	****	naïve vs. sucrose p = >0.9999, naïve vs. shock p < 0.0001, sucrose vs. shock p < 0.0001	naïve vs. sucrose z = 0.1148, naïve vs. shock z = 9.147, sucrose vs. shock z = 7.768	naïve vs. sucrose ns, naïve vs. shock ****, sucrose vs. shock ****
S.13	B	Canton-S shock-trained average preference index compared between odor off and post-choice	odor off W = 0.9216, choice W = 0.7955	odor off p = 0.0001, choice p <0.0001	N/A	N/A	Wilcoxon matched-pairs signed rank test	N/A	Canton-S shock-trained = 79, Canton-S sucrose-trained = 120, Dunce shock-trained = 76, Dunce sucrose-trained = 108,	< 0.0001	W = -1840	****	N/A	N/A	N/A
	C	Canton-S sucrose-trained average preference index compared between odor off and post-choice	odor off W = 0.9530, choice W = 0.9852	odor off p = 0.0004, choice p = 0.2126						0.0089	W = 1804	**			
	D	Dunce shock-trained average preference index compared between odor off and post-choice	odor off W = 0.9016, choice W = 0.9801	odor off p < 0.0001, choice p = 0.2727						0.0037	W = -1028	**			
	E	Dunce sucrose-trained average preference index compared between odor off and post-choice	odor off W = 0.9134, choice W = 0.9375	odor off p = 0.0025, choice p = 0.0174						0.2034	W = 544	ns			

Available for download at
https://journals.biologists.com/jeb/article-lookup/doi/10.1242/jeb.253164#supplementary-data

Journal of Experimental Biology • Supplementary information

Table S4. Fly and frame exclusions per group.

**Supplemental Table 4:** Data exclusion				
**Figure Panel**	**Genotype and Condition**	**Number and percent of flies excluded**	**Experimental replicates of arenas tested**	**Average frames excluded across lost-tracking events per-fly**
1B,C,D: S4B,C,D: S5B,C	**1:512 EA 1:512 IAAc Canton-S naïve**	2/120 (1.7%)	12	5.55/4050 (0.1%)
1B,C,D: S4B,C,D: S5D,E	**1:1024 EA 1:1024 IAAc Canton-S naïve**	11/80 (13.8%)	8	22.91/4050 (0.6%)
1B,C,D,F,L: 2A,B: 3A,B: 4B,I,P: S4B,C,D: S5F,G: S11A,C,D: S12A,G,H	**1:512 EA 1:1024 IAAc Canton-S naïve**	7/120 (5.8%)	12	7.22/4050 (0.2%)
1F,I,N: 2I,J: 3I,J: 4D,K,R,V,W: S7K-V: S8G-I: S11A,C,D: S12E,G,H	**Canton-S sucrose trained**	0/109	12	5.51/4050 (0.1%)
1F,H,N: 2E,F: 3E,F: 4C,J,Q,V,W: S7A-J: S8A-C: S11A,C,D: S12C,G,H	**Canton-S shock trained**	1/95 (1.1%)	11	11.99/4050 (0.3%)
1G,O: 2C,D: 3C,D: 4E,L,S: S5H,I: S11B,E,F: S12B,I,J	**1:512 EA 1:1024 IAAc W1118 naïve**	2/160 (1.3%)	16	6.18/4050 (0.2%)
1G,K,Q: 2K,L: 3K,L: 4G,N,U,X,Y: S8J-L: S11B,E,F: S12F,I,J	**W1118 sucrose trained**	1/96 (1%)	10	5.43/4050 (0.1%)
1G,J,P: 2G,H: 3G,H: 4F,M,T,X,Y: S8D,E,F: S11B,E,F: S12D,I,J	**W1118 shock trained**	44/216 (20.4%)	22	7.82/4050 (0.2%)
5B,G,H,I,J: S13D	**Dunce sucrose trained**	0/108	11	5/4050 (0.1%)
5A,C,D,E,F: S13C	**Dunce shock trained**	0/76	8	5.91/4050 (0.1%)
5B,G,H,I,J: S13B	**Canton-S sucrose trained (Dunce control)**	0/120	12	4.97/4050 (0.1%)
5A,C,D,E,F: S13A	**Canton-S shock trained (Dunce control)**	1/80 (1.3%)	8	6.01/4050 (0.1%)
S9B,C,N: S10A,B,C,G,H	**Canton-S odor-only control**	0/80	8	4.28/4050 (0.1%)
S9F,G,N: S10A,B,C,G,H	**Canton-S sucrose-only control**	0/80	8	4.78/4050 (0.1%)
S9J,K,N: S10A,B,C,G,H	**Canton-S shock-only control**	3/100 (3%)	10	5.17/4050 (0.1%)
S9D,E,N: S10D,E,F,G,H	**W1118 odor-only control**	14/80 (17.5%)	8	6.43/4050 (0.2%)
S9H,I,N: S10D,E,F,G,H	**W1118 sucrose-only control**	5/80 (6.3%)	8	5.16/4050 (0.1%)
S9L,M,N: S10D,E,F,G,H	**W1118 shock-only control**	6/100 (6%)	10	7.37/4050 (0.2%)

Available for download at
https://journals.biologists.com/jeb/article-lookup/doi/10.1242/jeb.253164#supplementary-data
